# Millettia speciosa reprograms the lung proteome and suppresses CCL24-driven eosinophilic inflammation in allergic asthma

**DOI:** 10.3389/falgy.2026.1726706

**Published:** 2026-06-03

**Authors:** Hangrui Yang, Geyuan Wu, Shuo Liang, Raona Zan, Yitao Chen, Weiwei Yuan, Hong Chen, Guiqiong Huang

**Affiliations:** 1 Guangzhou University of Chinese Medicine, Guangzhou, China; 2 Guangzhou University of Chinese Medicine, Huizhou Hospital, Huizhou, China

**Keywords:** asthma, CCL24, eosinophils, gut-Lung axis, millettia speciosa, proteomics, th2 inflammation

## Abstract

**Background:**

Asthma is a Th2-skewed inflammatory disorder characterized by eosinophilic infiltration, cytokine dysregulation, and airway remodeling. Emerging evidence highlights the role of immunometabolic pathways and the gut-lung axis in asthma pathogenesis.

**Methods:**

We investigate the therapeutic effects of Niudali (Millettia speciosa)**,** a traditional Chinese medicinal herb, in an ovalbumin-induced mouse model of allergic asthma using high-resolution data-independent acquisition (DIA) lung proteomics integrated with cytokine profiling.

**Results:**

Niudali treatment significantly alleviated airway inflammation and eosinophilic infiltration. Proteomic analysis revealed 179 differentially expressed proteins (DEPs), with a notable finding that CCL24, a key eosinophil-recruiting chemokine, was completely suppressed in Niudali-treated mice but highly expressed in the asthma model. This highlights the central role of CCL24 inhibition in the mechanism through which Niudali mitigates eosinophil-mediated inflammation.Functional enrichment analyses revealed that Niudali modulates pathways involved in complement and coagulation cascades, lipid transport, antioxidant defense, and PPAR signaling, reflecting a shift toward immune resolution and metabolic homeostasis. Network analysis identified key hub proteins, including Alb, Apoe, Apoa1, Proc, and Serpina7, which orchestrate lipid metabolism, antioxidant functions, and immune regulation. The modulation of serpins, apolipoproteins, and extracellular space-related proteins suggests a broad immunometabolic reprogramming effect. Notably, this molecular signature aligns with the gut-lung axis paradigm, potentially reflecting microbiota-mediated modulation via short-chain fatty acids (SCFAs). Consistent with proteomic findings, bronchoalveolar lavage fluid (BALF) analyses showed significant reductions in IgE, IL-4, IL-5, and IL-6**,** further confirming suppression of Th2-mediated inflammation.

**Conclusion:**

study provides proteomic evidence that Niudali treats asthma by disrupting the CCL24-eosinophil axis and rebalancing immunometabolic networks. These findings support Niudali as a promising candidate for gut-lung axis-targeted interventions in asthma and provide a systems-level framework for future microbiome metabolome integrated studies. While our findings suggest a potential link between these molecular changes and the gut-lung axis, this mechanism was not directly investigated in the present study and should therefore be considered hypothetical. Future studies incorporating microbiome and metabolomic analyses will be essential to clarify the role of gut-derived metabolites, including SCFAs, in mediating these effects.

## Graphical Abstract

**Figure:**
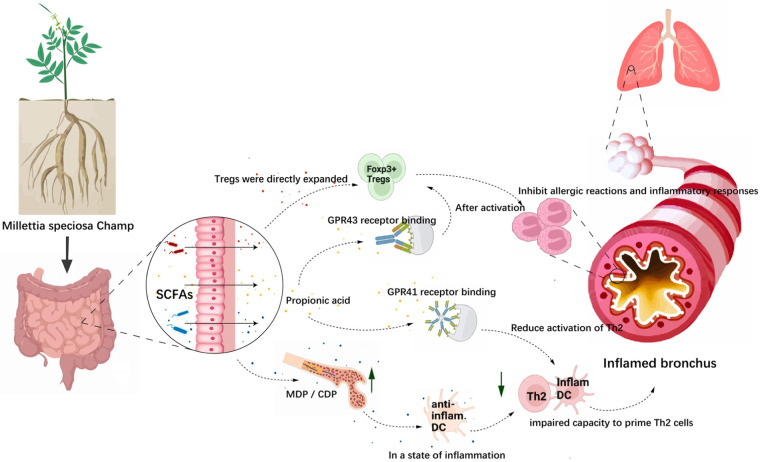

## Introduction

Asthma is a chronic, heterogeneous respiratory disease affecting more than 260 million people globally (23), with a growing burden in China, particularly among children (1–4). Eosinophilic asthma, a common endotype, is characterized by Th2-driven inflammation mediated by IL-4, IL-5, and IL-13, alongside the recruitment of eosinophils via chemokines such as CCL24 (eotaxin-2) (5, 6). CCL24 promotes eosinophil migration through CCR3 signaling and acts synergistically with IL-5 to amplify IL-13 production and airway hyperresponsiveness (AHR) (5–7). Recent studies have shown that eosinophil extracellular traps (EETs) further amplify this loop by stimulating epithelial CCL24 secretion and activating ILC2s, exacerbating Th2 inflammation and airway remodeling (8, 9). This pathogenic axis contributes to steroid-resistant asthma phenotypes and chronic airway fibrosis (10). Despite advances in biologics, asthma control remains suboptimal in many regions, including China, where environmental factors such as pollution, dietary changes, and urbanization worsen disease severity (1, 3, 4). The gut–lung axis has emerged as a key regulator of airway immunity. Microbiota-derived short-chain fatty acids (SCFAs) suppress Th2 cytokines, enhance regulatory T cells, and maintain epithelial integrity (11). This aligns with traditional Chinese medicine (TCM) concepts, where gut health is fundamental to respiratory resilience.

Millettia speciosa (Niudali), a TCM herb known for enhancing gut barrier function and modulating the microbiota, exhibits anti-inflammatory, antioxidant, and immunomodulatory properties (12–14). However, direct molecular evidence of its effects on allergic airway inflammation remains unexplored. Meanwhile, in the theory of TCM, asthma is considered a disease caused by a variety of factors, which involve dysfunctions of the zang - fu organs, qi disorders, and other issues. Its primary disease locations are the lungs, spleen, and kidneys. Therefore, TCM holds that the treatment of asthma requires a comprehensive regulation of the functions of the internal organs in the body. Common treatment methods include “tonifying the lungs and strengthening the kidneys” and “strengthening the spleen and regulating qi”. Pingcyangguben, a traditional Chinese medicine prescription commonly used in the treatment of asthma, has the effects of tonifying the lungs and securing the kidneys, containing qi and relieving asthma, supplementing qi and strengthening the spleen, relieving cough and eliminating phlegm. It treats the three zang organs to achieve the cause treatment, and treating cough, phlegm and asthma to achieve symptomatic treatment. Niudali, as a traditional Chinese medicine, also has the effects of tonifying the lungs, strengthening the spleen, and tonifying the kidneys. We selected Pingcyangguben as a control drug mainly because it has a similar mechanism to Niudali in TCM treatment. Thus, it can assist us in understanding the efficacy of Niudali in the treatment of asthma compared with other traditional Chinese medicines based on the same TCM theory.

Here, we hypothesize that Niudali mitigates allergic asthma by suppressing eosinophilic chemotactic signals, particularly CCL24, and restoring immunometabolic balance. Using high-resolution DIA-based lung proteomics combined with cytokine validation, we aimed to characterize how Niudali rewires the lung proteome, focusing on immune modulation, lipid metabolism, and oxidative stress pathways.

## Methods

### Extraction and preparation of traditional Chinese herbal medicines

Niudali was extracted through water reflux extraction and then freeze-dried into powder. Three hundred and sixty grams of Niudali were crushed into coarse powder. For the first extraction, 10 times the amount of water was added, followed by a 30 - minute soaking period, a 60 - minute heat reflux process, and then filtration. Subsequently, 8 times the amount of water was added to the filter residue, and it was heated under reflux for 40 min. The filtrates were combined and concentrated to 500 mL under reduced pressure at 65 °C. The concentrated solution was freeze-dried for 72 h and was ready for use.

Pingcyangguben contains more than 20 Chinese herbs, such as Astragalus membranaceus, Atractylodes atractylodes, windstorm, Poria, etc. It is decocted and concentrated to a paste in the Chinese Medicine Pharmacy of Huizhou Hospital, Guangzhou University of Chinese Medicine. The dose of the mice was as follows: high dose group: 8.32 g/kg was given by 10 mL/kg to obtain 0.832 g/mL, and 41.6 g was taken and fixed in 50 mL pure water; Low dose group: 4.16 g/kg was given by 10 mL/kg to get 0.416 g/mL, 18.72 g was fixed to 45 mL with pure water.

### Animals and experimental design

Female BALB/c mice (6–8 weeks old) were maintained under specific pathogen-free conditions. All experimental protocols were approved by the institutional animal care and use committee. Allergic airway inflammation was induced via ovalbumin (OVA) (24). The mice were randomly divided into two groups (*n* = 3 per group): the OVA-induced model group and the Niudali-treated group (OVA + oral *Millettia speciosa* extract). Oral administration of Niudali or Montelukast was performed during the challenge phase (25), and lung tissues were collected for proteomic analysis after the final challenge.

### Pathological examination of mouse lung tissue

The mice were euthanized 24 h after the final challenge. Lung tissues were fixed in a 4% paraformaldehyde solution for 24 h, subsequently embedded, and sectioned at a thickness of 4 μm. Following dewaxing, the tissue sections were subjected to hematoxylin and eosin (HE), periodic acid-Schiff (PAS), and Masson's trichrome staining on separate slides. The slides were dehydrated via various alcohol gradients, sealed with a neutral resin, and examined under a microscope for analysis and documentation.

### Protein extraction and quantification

For proteomic extraction analysis, the high-dose Niudali group was selected because it exhibited the most pronounced therapeutic effects on airway inflammation and eosinophilic infiltration. Due to the limited number of samples and the high cost associated with high-resolution DIA proteomics, only this dose was analyzed. The lung tissues were immediately snap-frozen in liquid nitrogen and ground using a cryogenic grinder. The samples were lysed in buffer containing PMSF and phosphatase inhibitors. The lysates were subsequently centrifuged at 12,000 rpm for 10 min at 4 °C, after which the supernatants were collected. Protein concentrations were measured via the bicinchoninic acid (BCA) assay according to the manufacturer's instructions. A standard curve was generated via the use of serial dilutions of bovine serum albumin (BSA), and the absorbance was read at 562 nm. Protein integrity was verified by loading 10 μg of protein per sample on a 12% SDS‒PAGE gel, followed by Coomassie Brilliant Blue staining via the eStain LG system and digital imaging.

### Trypsin digestion and peptide desalting

Equal amounts of protein were reduced with 5 mM dithiothreitol (DTT) at 55 °C for 30 min and alkylated with 10 mM iodoacetamide (IAA) in the dark at room temperature. Proteins were precipitated with six volumes of cold acetone and incubated at −20 °C overnight. The pellets were redissolved in 50 mM ammonium bicarbonate and digested with trypsin (1:50, w/w) at 37 °C overnight. The reactions were terminated by acidification with formic acid (final pH ∼3). The peptides were desalted via SOLA™ C18 extraction plates, eluted with 50% acetonitrile containing 0.1% formic acid, dried under vacuum, and stored at −80 °C.

### LC–MS/MS data acquisition

Desalted peptides were reconstituted and spiked with indexed retention time (iRT) standards (Biognosys) at a 1:20 ratio. The samples were analyzed via a nano-UHPLC system (U3000, Thermo Fisher) equipped with a C18 column (15 cm × 75 μm, 1.6 μm). The LC gradient consisted of a linear increase from 5% to 80% buffer B (acetonitrile with 0.1% formic acid) over 30 min at a flow rate of 400 nL/min. Mass spectrometry was performed on a timsTOF Pro instrument (Bruker) operating in Data Independent Acquisition (DIA) mode. The key acquisition parameters included a capillary voltage of 1.4 kV, dry gas at 3.0 L/min, dry temperature of 180 °C, mass range of 100–1700m/z, and ion mobility between 0.7–1.3. The collision energies ranged from 20 to 59 eV.

### DIA data processing and quantification

The raw DIA data were processed via Spectronaut Pulsar (version 18.4, Biognosys) with the UniProt *Mus musculus* FASTA database (10090, version 2024.2.1). The search parameters included trypsin/*P* as the enzyme, allowance for two missed cleavages, carbamidomethylation (C) as a fixed modification, and oxidation (M) and N-terminal acetylation as variable modifications. The precursor and protein Q value cutoffs were both set at 0.01. Protein quantification was based on MS2-level data, and identifications were filtered to remove contaminants and decoys. Protein abundances were log-transformed and normalized prior to statistical analysis.

### Bioinformatic and statistical analysis

Differentially expressed proteins were identified via unpaired t tests with thresholds of *p* < 0.05 and a fold change ≥ 1.5 or ≤ 0.667. Principal component analysis (PCA), volcano plots, and unsupervised hierarchical clustering were performed to assess global proteomic variation. Functional annotation and enrichment analyses were conducted via the Gene Ontology (GO), KEGG, Reactome, InterPro, Pfam, eggNOG, and WikiPathways databases. Differentially expressed proteins were further examined via gene set enrichment analysis (GSEA), subcellular localization prediction, and protein–protein interaction (PPI) network construction. The visualization tools used included heatmaps, bar graphs, bubble plots, chord diagrams, and Venn diagrams. All figures and extended datasets are provided in the Supplementary Information.

## Results

### Effects of niudali on pathological changes in the lung tissue of asthmatic mice

**Figure 1:**
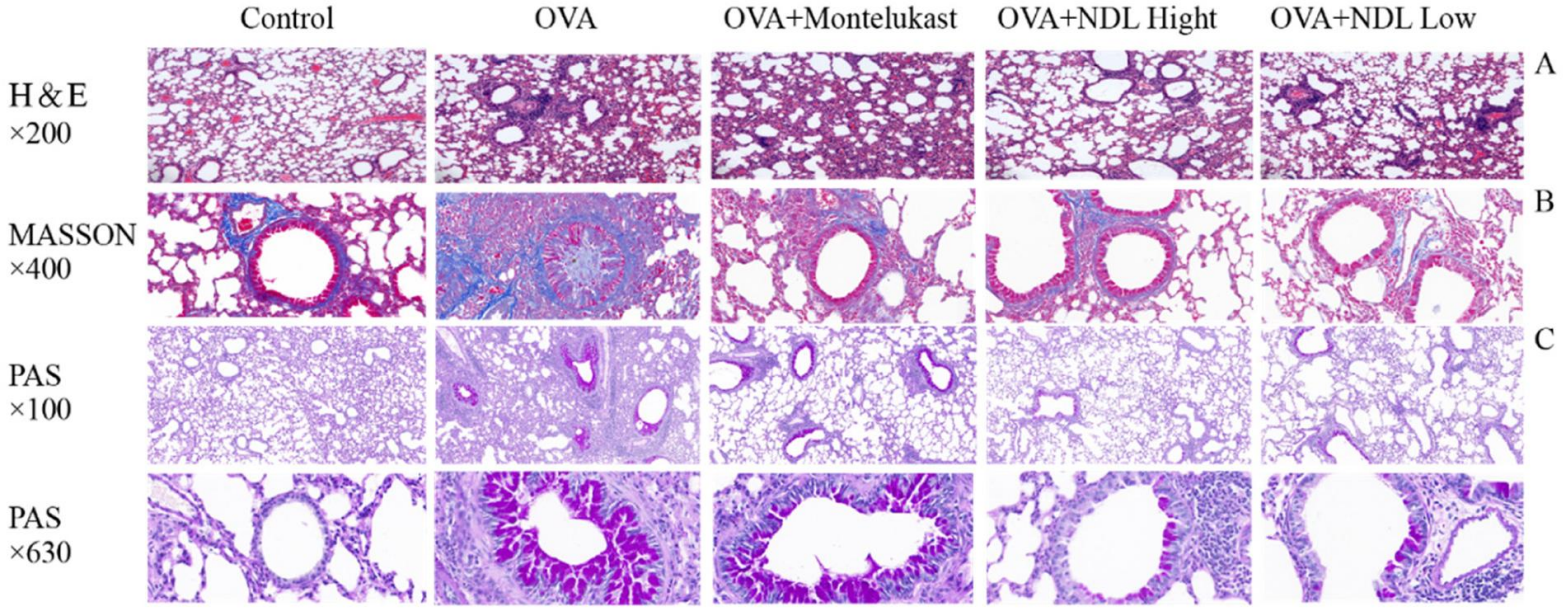
The impact of Millettia speciosa (niudali) on lung tissue pathology in asthmatic mice was investigated. **A)**Lung tissue pathology images from the OVA model group revealed increased immune cell infiltration around the trachea, thickening of alveolar walls, alveolar collapse, and scattered distribution of various cells in the lungs. Treatment with NDL effectively ameliorated these conditions. **B)** The OVA group exhibited a significant presence of blue collagen fibers in alveolar septa and inner alveolar walls. While the montelukast sodium group mitigated collagen fiber accumulation, it led to diffuse alveolar dilation, alveolar septal breakage, and increased alveolar fusion. **C)** Enhanced mucus secretion was observed in the bronchi of the OVA group. Treatment with NDL significantly reduced tracheal mucus secretion.

The control group of mice presented an intact lung tissue structure with no damage, whereas the model group of mice presented severe lung tissue damage, including inflammatory cell infiltration, goblet cell proliferation, mucus secretion, and thickened airway walls. In the low-dose and high-dose Niudali groups, improvements in alveolar wall thickening and collapse, reduced inflammatory infiltration, and thinner airway walls were observed (Figure 1A). Compared with that in the control group, Masson staining revealed increased collagen fiber proliferation in the bronchial walls of the model group. The Montelukast treatment had no significant effect on collagen fibers in asthmatic mice, whereas the low and high dose Niudali treatments significantly inhibited collagen fiber proliferation (Figure 1B). PAS staining revealed purplish-red goblet cells and mucus in the bronchi. The control group showed no PAS staining in the lung bronchioles, whereas the model group presented purplish red-stained goblet cells and mucus (Figure 1C). These results suggest that Niudali effectively reduces goblet cell secretion in asthmatic mice, outperforming Montelukast. Niudali is indicated as an effective asthma treatment.

### High-Quality proteomic profiling enables deep characterization of lung tissue

**Figure 2:**
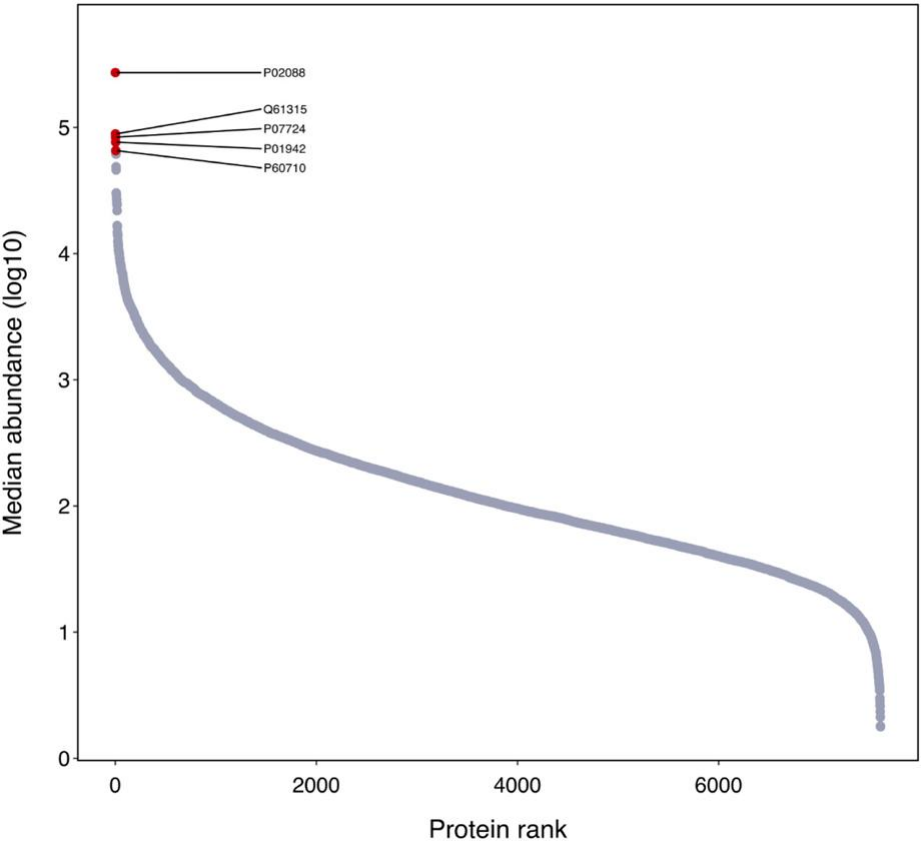
Rank abundance distribution highlights core lung proteins with dominant expression profiles.rank abundance curve of lung proteins, with the *x*-axis representing protein rank (ordered from highest to lowest abundance) and the *y*-axis showing median protein abundance on a log₁₀ scale. Most proteins cluster between log₁₀ values of 2–4, reflecting a balanced detection range. A subset of highly abundant proteins is marked in red.

We achieved deep proteomic coverage of lung tissue samples, identifying thousands of proteins with high confidence. The molecular weight distribution analysis revealed that most identified proteins ranged between 20 and 60 kDa, with the highest counts observed in the 20–30 kDa (1,014 proteins), 30–40 kDa (981 proteins), and 40–50 kDa (904 proteins) bins. A substantial number of proteins were also detected in higher molecular weight ranges, including 286 proteins above 200 kDa, highlighting the robustness of the analytical workflow in capturing a broad molecular spectrum (Supplementary Figure S1). Protein abundance analysis, ranked by median abundance on a log10 scale, demonstrated that the majority of proteins were distributed between log10 values of 2 and 4, indicating a balanced dynamic range of detection. Notably, a small set of highly abundant proteins, including P02088 (Hbb-b1), Q61315 (Apc), P07724 (Alb), P01942 (Hba), and P60710 (Actb), presented log10 values exceeding 5, indicating their dominant presence in lung tissue proteomes (Figure 2). These proteins represent essential components of lung physiology, hemoglobin subunits (Hbb-b1, Hba) for oxygen transport, Alb for maintaining osmotic balance and molecular transport, Actb as a key cytoskeletal protein, and Apc, a regulator of epithelial homeostasis via Wnt signaling. Their prominence aligns with the cellular architecture and physiological demands of normal mouse lung tissue (15), further supporting the reliability and biological validity of our dataset. The peptide length distribution was consistent with the expected tryptic digestion patterns, with the majority of peptides ranging from 9 to 11 amino acids, peaking at 10 amino acids (13,440 peptides), followed closely by 9 (11,172 peptides) and 11 (10,727 peptides) (Supplementary Figure S2), supporting efficient digestion and peptide recovery. Additionally, analysis of peptide coverage per protein revealed that a substantial proportion of proteins were identified with high redundancy; specifically, 2,165 proteins were supported by more than 20 unique peptides, underscoring the depth, confidence, and reproducibility of this proteomic dataset (Supplementary Figure S3).

### Robust data normalization and distinct proteomic signatures differentiate niudali-treated mice from asthmatic mice

Comprehensive data quality assessments confirmed excellent normalization, high technical reproducibility, and biological consistency across samples. Density distribution plots demonstrated that protein intensities were unimodal and approximately normally distributed across all biological replicates and treatment groups, indicating effective normalization without systematic bias (Supplementary Figure S4). This finding indicates that systematic biases, such as batch effects or technical artifacts, were effectively minimized during normalization.

To further evaluate the consistency of the quantitative measurements within each group, we examined the relative standard deviation (RSD) of the protein intensities. While density plots assess global distribution alignment, RSD provides a direct measure of variability among biological replicates within groups. The results revealed median RSD values less than 0.2 across all groups, indicating high technical reproducibility and minimal within-group variation (Supplementary Figure S5). While RSD quantifies the spread of protein intensities within groups, it does not capture the overall similarity of proteomic profiles across samples. To complement this, we performed Pearson correlation analysis, which evaluates the linear relationship between entire proteome profiles of individual samples. Consistent with the low RSD values, the correlation matrix showed strong intragroup similarity, with correlation coefficients exceeding r > 0.90 within both the Niudali-treated and asthma model groups (Supplementary Figure S6). This further confirms that biological replicates not only are low in variability but also maintain highly similar global protein expression patterns.

**Figure 3:**
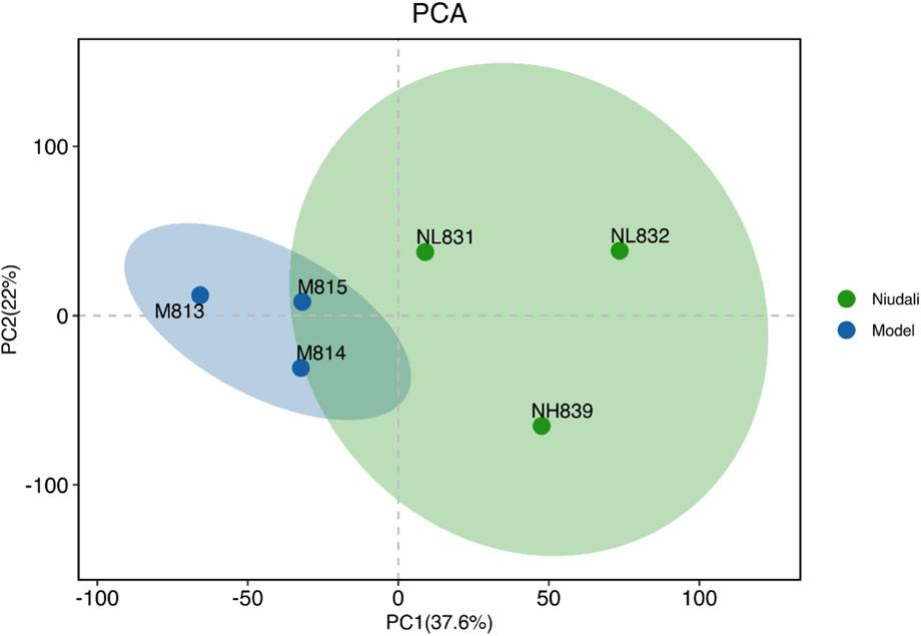
Principal component analysis reveals distinct proteomic signatures between niudali-treated and model groups. Principal Component Analysis (PCA) plot showing global proteomic variation between Niudali-treated mice (green) and model group mice (blue). The *x*-axis (PC1) explains 37.6% of the total variance, capturing the largest source of variation, while the *y*-axis (PC2) explains 22%, representing the second-largest variance component. Each point represents an individual sample, with shaded ellipses denoting 95% confidence intervals for each group. The project conducted experiments or analyses using nine samples, comprising three experimental groups and two control groups. The sample IDs and corresponding groups used in the experiment. The specific sample IDs for each group are listed below: Model group: M813, M814, M815 (3 samples); Niudali group: NL832, NL831, NH839 (3 samples); Pingcyanguben group: PH852, PL861, PH855 (3 samples).

Importantly, principal component analysis (PCA) revealed clear proteomic separation between the asthma model group and the Niudali-treated group, with replicates clustering tightly within each group (Figure 3). The first two principal components explained 37.6% (PC1) and 22% (PC2) of the total variance, capturing the dominant proteomic differences driven by disease vs. treatment. This distinct segregation underscores that Niudali treatment induces systematic remodeling of the lung proteome, separating it from the asthmatic state and reflecting a unique therapeutic proteomic signature. Together, these layers of validation, from distribution assessment to variability checks and global correlation, culminating in multivariate clustering, demonstrate that the dataset is both technically robust and biologically meaningful, providing a reliable foundation for downstream differential proteomic analysis.

### Niudali induces a distinct anti-inflammatory and redox-regulating proteomic signature in the inflamed lung microenvironment

**Figure 4:**
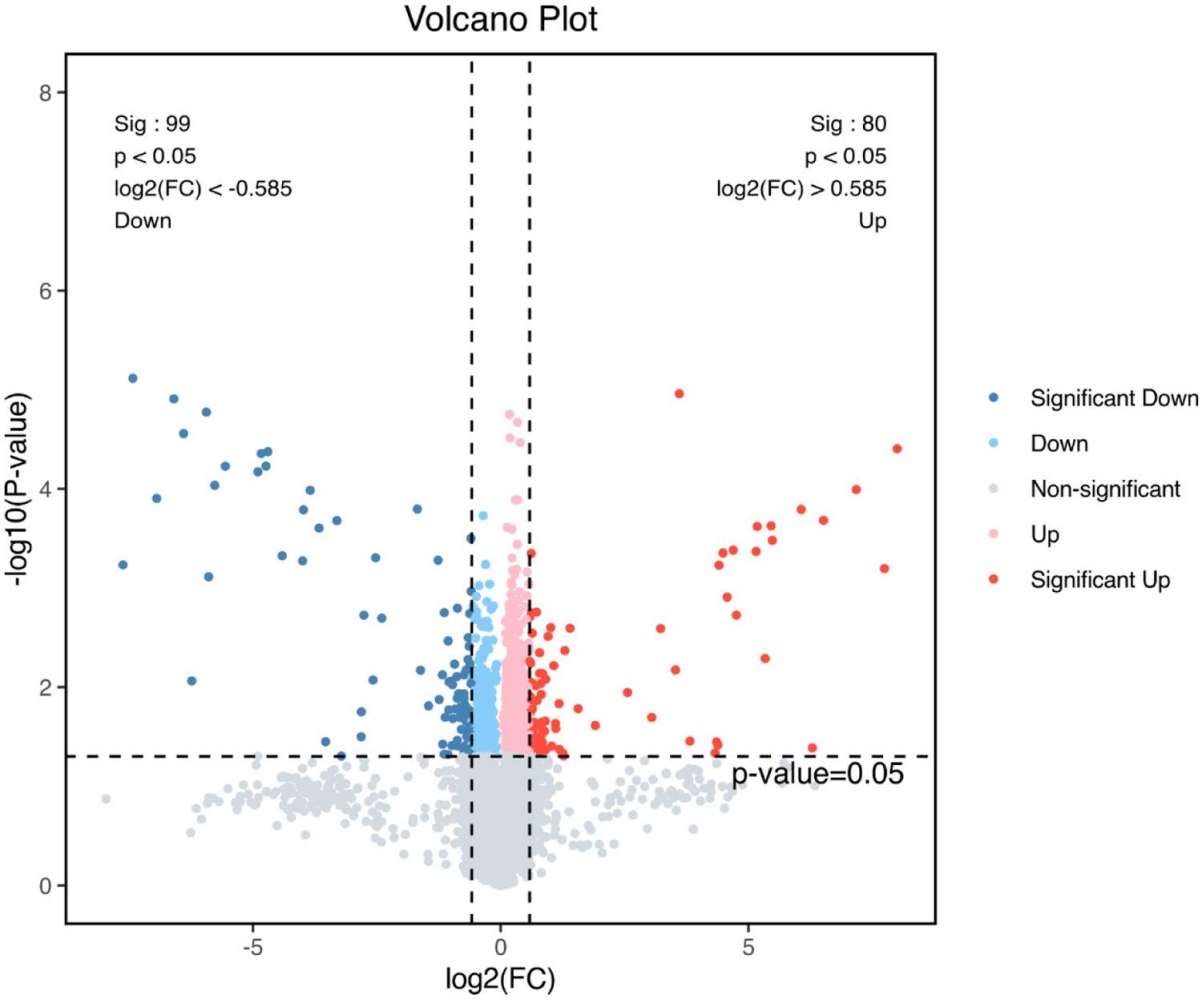
Volcano plot reveals DEPs in niudali-treated vs. Asthmatic Mice.Volcano plot showing the distribution of differentially expressed proteins based on statistical significance and fold change. The *x*-axis represents log₂ fold change (log₂FC), where positive values indicate upregulation and negative values indicate downregulation in the Niudali group compared to the model group. The *y*-axis represents the –log₁₀(*p*-value), indicating statistical significance. Horizontal dashed line marks the *p*-value threshold (*p* = 0.05), and vertical dashed lines indicate the fold-change cutoff (log₂FC = ±0.585). Colored dots represent protein groups: red for significantly upregulated, blue for significantly downregulated, pink and light blue for non-significant fold changes with *p* < 0.05, and gray for non-significant proteins (*p* > 0.05).

Following the confirmation of strong data integrity and clear group-level separation, we next investigated the specific proteomic alterations induced by Niudali treatment. Volcano plot analysis revealed a total of 179 differentially expressed proteins (DEPs) when the Niudali-treated group was compared with the asthma model group (Figure 4). Among these proteins, 99 were significantly downregulated and 80 were significantly upregulated in the Niudali group, as indicated by thresholds of log₂-fold change > 0.585 and *p* < 0.05. Notably, several key inflammatory and fibrosis-associated proteins, including complement factor H (P06909), serum amyloid A1 (Q07008), and periostin (Q62009), were among the most strongly suppressed. These proteins are closely linked to fibrotic remodeling, complement activation, and acute-phase inflammatory responses, suggesting that Niudali may attenuate key pathological processes associated with allergic airway inflammation. On the other hand, proteins involved in immune regulation and redox homeostasis, such as the transferrin receptor (Q62351) and glutathione peroxidase 1 (P11352), were significantly upregulated, suggesting a potential role of Niudali in enhancing antioxidant defenses and immune resolution mechanisms.

**Figure 5:**
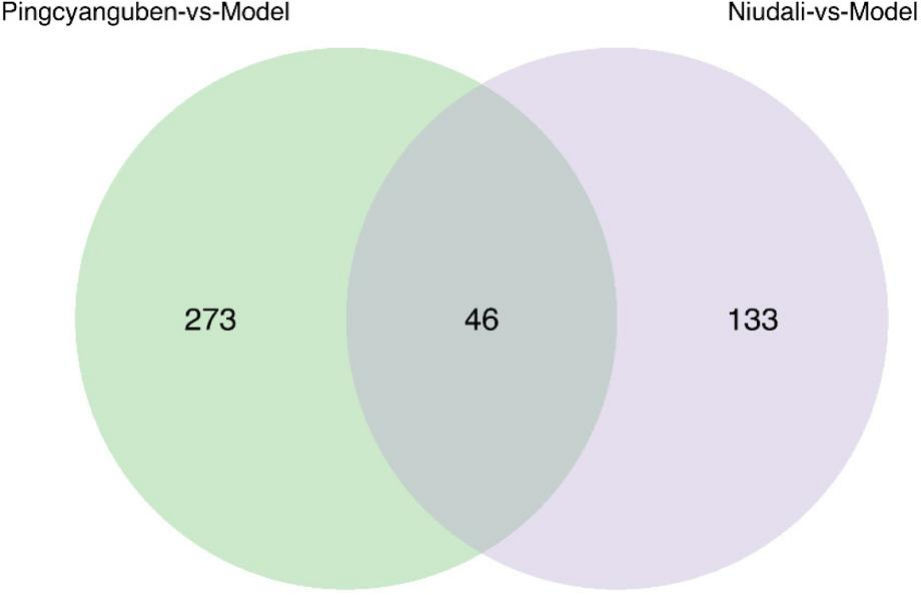
Venn diagram reveals 133 unique proteins modulated by niudali. Venn diagram comparing differentially expressed proteins (DEPs) between Niudali vs. Model (right circle) and Pingyangguben vs. Model (left circle). The numbers represent the unique and shared DEPs between the two treatments. Niudali exhibits 133 unique DEPs, Pingyangguben has 273 unique DEPs, and 46 DEPs are common to both treatments.

To further assess whether the proteomic signature of Niudali is distinct from that of the other treatments, we performed a comparative Venn diagram analysis against the proteomic changes induced by Pingyangguben treatment (Figure 5). This analysis revealed that 133 DEPs were uniquely regulated by Niudali, 273 were specific to Pingyangguben, and 46 DEPs were shared between the two treatments. This substantial set of Niudali-specific DEPs highlights that Niudali elicits a distinct molecular response, differentiating its mode of action from that of Pingyangguben, particularly in the modulation of inflammation, immune regulation, and oxidative stress pathways.

### Niudali selectively suppresses CCL24 and eosinophil-driven inflammatory pathways

**Figure 6:**
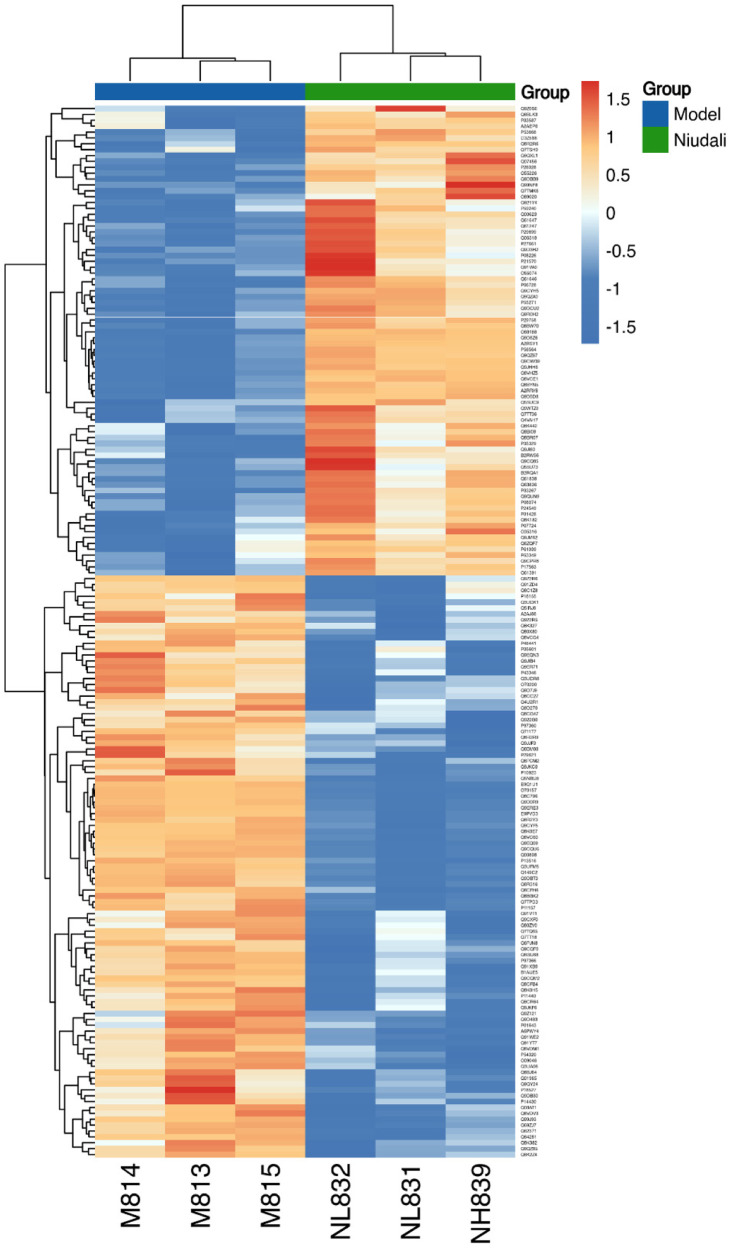
Heatmap of differentially expressed proteins highlights distinct proteomic profiles between niudali-treated and asthmatic mice. Hierarchical clustering heatmap displaying the expression patterns of differentially expressed proteins (DEPs) between Niudali-treated (green) and model (blue) groups. Rows represent individual proteins, and columns represent biological replicates. The color scale indicates Z-score normalized expression, ranging from low (blue) to high (red) abundance. Distinct clustering patterns demonstrate clear separation between the two groups based on their proteomic profiles.

The heatmap of differentially expressed proteins (DEPs) clearly illustrates a distinct proteomic separation between the Niudali-treated group and the asthma model group, highlighting widespread shifts in protein expression profiles following treatment (Figure 6). A prominent feature observed in the heatmap is the robust suppression of a cluster of inflammation-associated proteins in the Niudali group relative to the model group. Among these genes, CCL24 (Q9JKC0), also known as C-C motif chemokine 24 or eotaxin-2, was entirely undetectable in all the Niudali-treated samples but remained highly expressed in the model group. As a potent chemoattractant for eosinophils and an activator of M2 macrophage-mediated tissue remodeling, CCL24 plays a pivotal role in sustaining eosinophilic inflammation and airway remodeling characteristic of allergic asthma. Its complete suppression strongly suggests that Niudali effectively interrupts eosinophil recruitment and the associated inflammatory cascade.

In addition to CCL24, the heatmap highlights the marked downregulation of several other key proteins with functional relevance to asthma pathology. Aquaporin-4 (Aqp4, A2AQP0), a water channel protein involved in maintaining airway surface hydration and associated with inflammation-driven airway edema, was significantly suppressed in the Niudali group, which may have contributed to reduced mucus hypersecretion and airway hyperresponsiveness. Similarly, cadherin-related family member 5 (Cdhr5, Q8BGK2), an epithelial cell adhesion molecule linked to barrier function, is downregulated, suggesting a reduction in inflammation-induced epithelial remodeling and dysfunction. Furthermore, fibronectin 1 (Fn1, O70157), a critical extracellular matrix protein involved in fibrosis and airway structural remodeling, is also notably suppressed, indicating that Niudali may mitigate fibrotic processes within the lung.

Together, the patterns displayed in the heatmap reflect a coordinated anti-inflammatory and antifibrotic response elicited by Niudali treatment. The simultaneous suppression of eosinophil chemotaxis, mucosal edema factors, epithelial remodeling proteins, and fibrotic matrix components underscores the comprehensive nature of the therapeutic action of Niudali in resolving allergic airway inflammation and restoring lung homeostasis.

### Functional enrichment and network analysis reveal Key immunometabolic regulators in niudali-treated lungs

**Figure 7:**
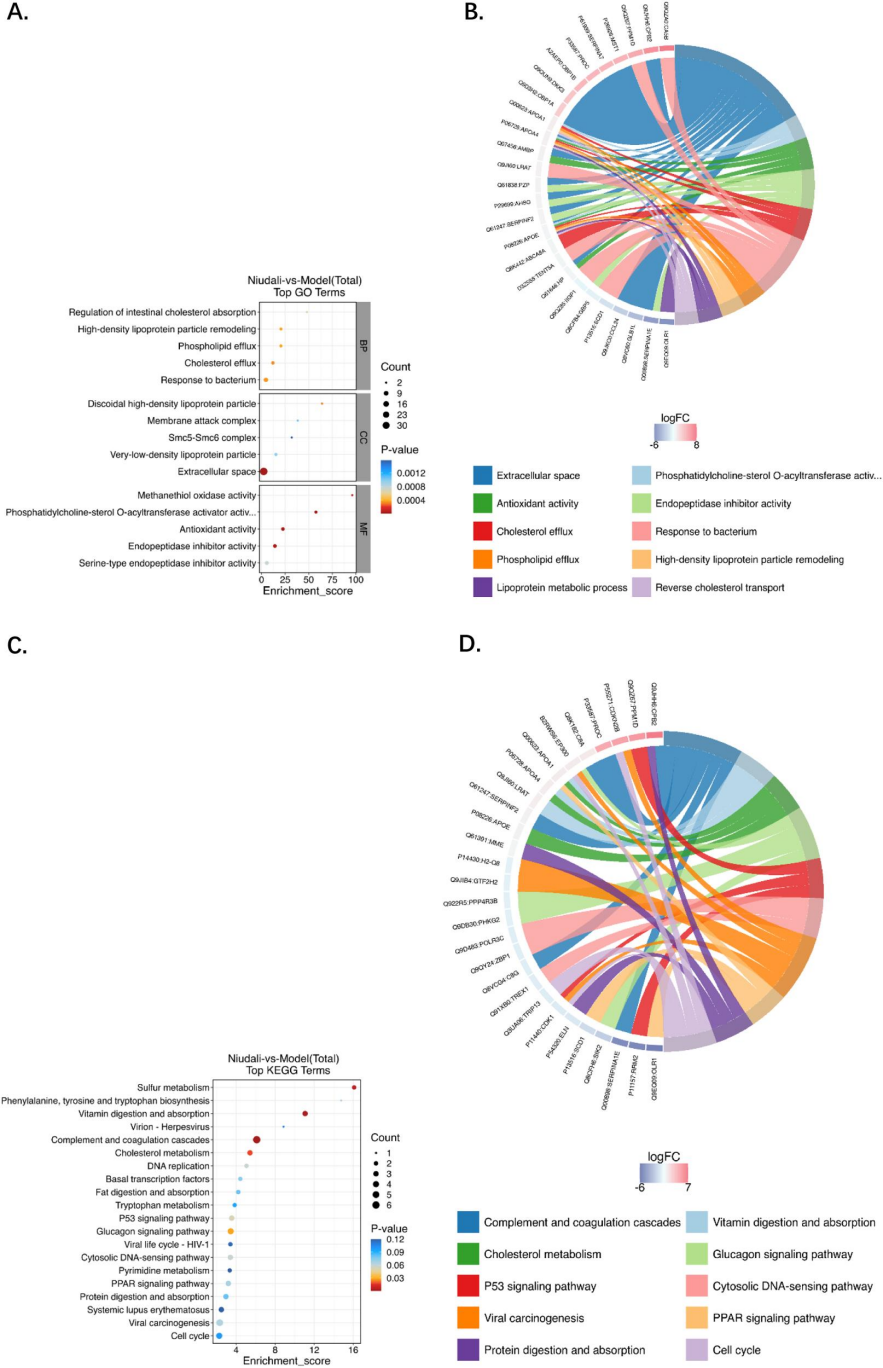

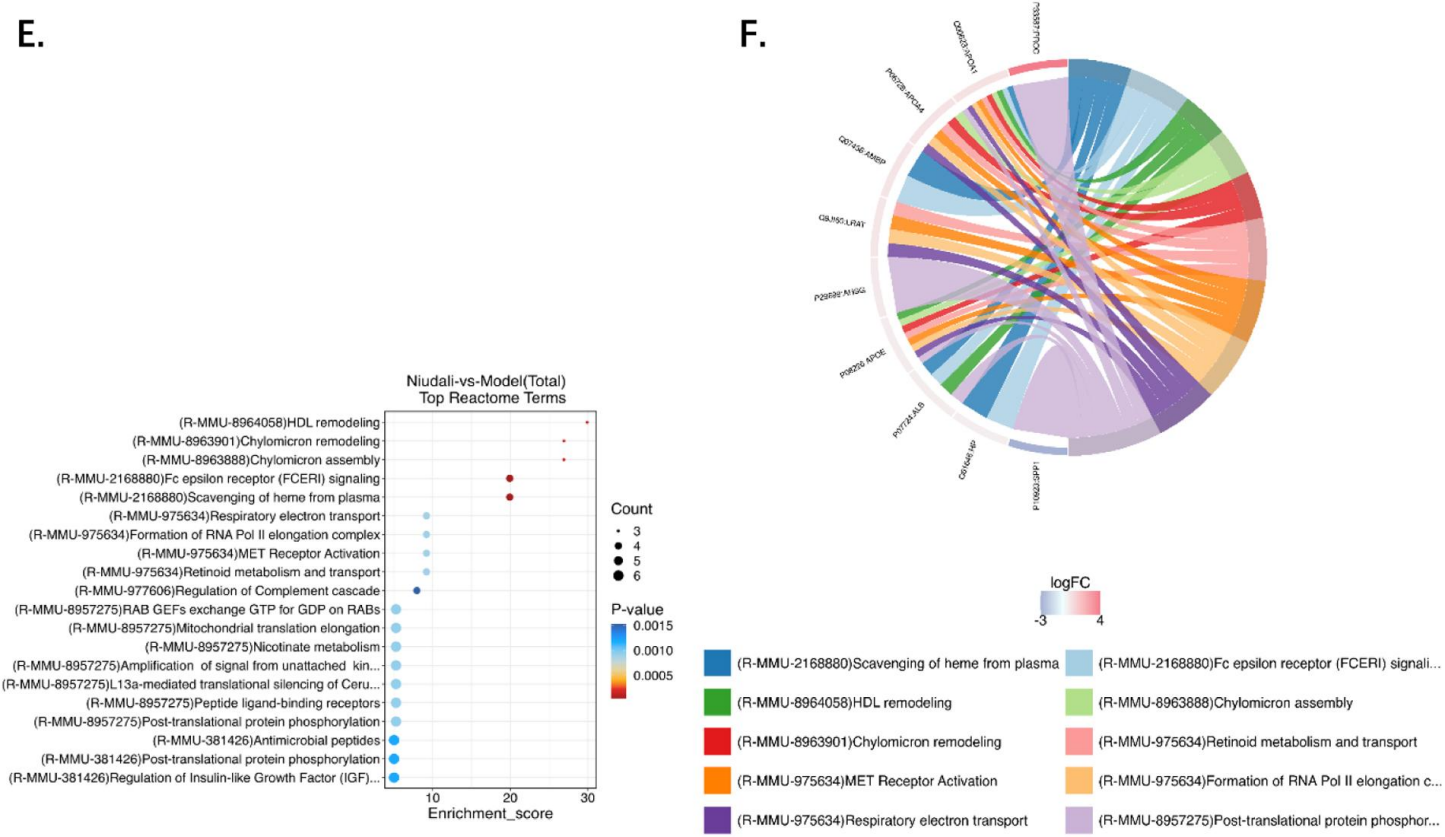
Functional enrichment analysis reveals lipid metabolism, antioxidant defense, and immune modulation in niudali-treated asthmatic lungs. **(A)** Bubble plot of the top enriched Gene Ontology (GO) terms, categorized into Biological Process (BP), Cellular Component (CC), and Molecular Function (MF). The *x*-axis indicates enrichment scores, bubble size represents the number of proteins, and color reflects *p*-values, with warmer colors indicating higher significance. **(B)** Chord plot visualizing key differentially expressed proteins (DEPs) contributing to multiple GO terms, highlighting their multifunctional roles. **(C,D)** KEGG pathway enrichment plots show top pathways related to immune regulation and metabolic processes. **(E,F)** The Reactome pathway analysis bubble plot **(E)** and corresponding chord diagram **(F)** display major pathways influenced by Niudali, including HDL remodeling, Fc*ε*RI signaling, complement regulation, and metabolic remodeling.

To gain mechanistic insights into the biological processes modulated by Niudali treatment, we performed Gene Ontology (GO) enrichment analysis of the differentially expressed proteins (DEPs). The top enriched biological processes (BP) included lipid transporter activity, high-density lipoprotein (HDL) remodeling, cholesterol efflux, antioxidant activity, phospholipid efflux, and immune-related responses such as response to bacteria (Figure 7A). The CC terms were dominated by extracellular space, membrane attack complex, and lipoprotein particles, reflecting the secretory and extracellular natures of the many lung proteins affected. Molecular function (MF) terms further supported the involvement of phosphatidylcholine-sterol O-acyltransferase activator activity, endopeptidase inhibitor activity, and antioxidant functions, highlighting the intersection of lipid metabolism and immune regulation. The chord plot (Figure 7B) clearly illustrates the contributions of key DEPs, such as Apoa1, Apoe, Alb, Pzp, and Ambp, across multiple enriched terms, reinforcing their central involvement in lipid handling and immune modulation.

Consistent with the GO results, the KEGG pathway analysis further highlighted significant enrichment in complement and coagulation cascades, cholesterol metabolism, fat digestion and absorption, and PPAR signaling pathways (Figures 7C,D). These pathways underscore the therapeutic effects of both the immune response and metabolic regulation by Niudali. Reactome pathway analysis confirmed these findings, revealing enrichment in pathways related to HDL remodeling, chylomicron assembly and remodeling, Fc epsilon receptor (Fc*ε*RI) signaling, scavenging of heme, respiratory electron transport, and regulation of the complement cascade (Figures 7E,F). Collectively, these results indicate that Niudali exerts multifaceted biological effects that span lipid metabolism, oxidative stress reduction, and inflammatory resolution, processes that are highly relevant in allergic airway inflammation and lung immune homeostasis.

**Figure 8:**
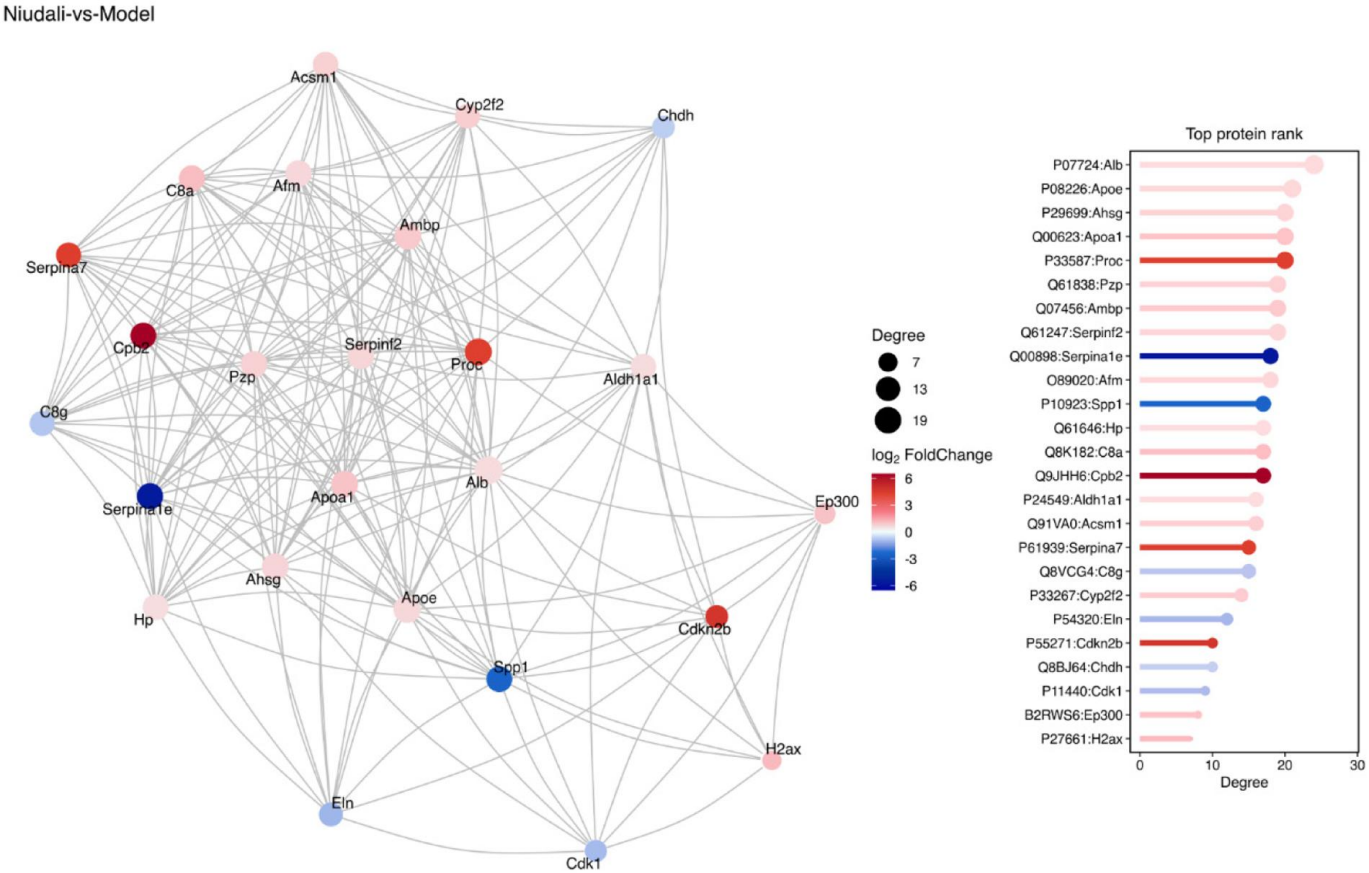
Protein-Protein interaction network highlights Key immunometabolic hubs in niudali-treated asthmatic lungs. Protein–protein interaction (PPI) network of differentially expressed proteins (DEPs) between the Niudali-treated and asthma model groups. Node size reflects the degree (number of connections), and node color indicates log₂ fold change (red for upregulated, blue for downregulated proteins). Highly connected hub proteins such as Alb, Apoe, Proc, Serpina7, and Serpinf2 are central to the network, representing regulators of lipid metabolism, coagulation, and immune responses. The right panel displays a ranked bar plot of the top proteins based on network degree, providing a quantitative assessment of their centrality within the interaction network.

While the functional enrichment analyses provided detailed insights into *which biological processes and pathways* are modulated, they did not fully capture *which proteins act as key regulatory hubs orchestrating these changes*. To address this, we performed protein–protein interaction (PPI) network analysis, which offers a systems-level view of how proteins interact and coordinate cellular responses. This step is critical because biological outcomes are not solely determined by individual proteins but also arise from the collective dynamics of interconnected protein networks. The PPI network identified Alb (albumin), Apoe (apolipoprotein E), Proc (protein C), Serpina7 (thyroxine-binding globulin), and Serpinf2 (alpha-2-antiplasmin) as key hub proteins with the highest degree of connectivity (Figure 8). These proteins are central regulators of immune modulation, coagulation balance, and lipid transport, underscoring their pivotal role in the immunometabolic reprogramming induced by Niudali. The domain analysis further revealed enrichment of serpins, lipocalins, and lipid-binding protein families, suggesting that the therapeutic effect of Niudali is driven by coordinated suppression of proinflammatory signals and enhancement of lipid-driven immune regulation.

### Niudali alleviates airway inflammation by suppressing BALF cytokines and IgE levels in asthmatic mice

**Figure 9:**
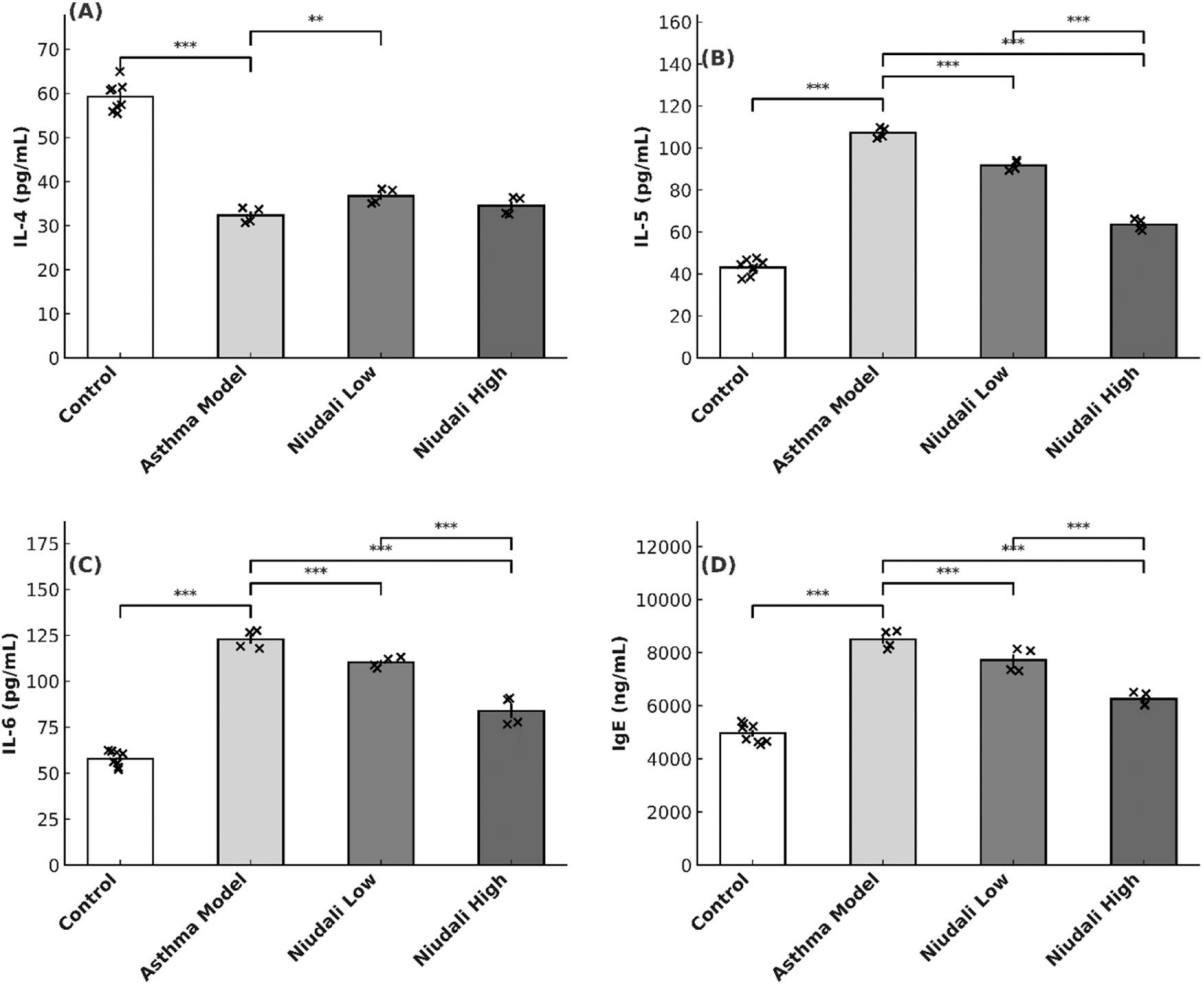
Niudali reduces type 2 cytokines and IgE levels in BALF of asthmatic mice. Quantification of bronchoalveolar lavage fluid (BALF) cytokines and IgE levels across experimental groups. (**A**) The plots display the concentrations of IL-4 levels (pg/mL) in Control, Asthma Model, Niudali Low, and Niudali High groups. (**B**) IL-5 levels (pg/mL) in the same groups. (**C**) IL-6 levels (pg/mL) in the same groups. (**D**) IgE levels (ng/mL) in the same groups. The *y*-axis represents protein concentration (pg/mL for cytokines and ng/mL for IgE), while the *X*-axis represents treatment groups. Bars indicate mean ± standard error (SE). Statistical significance was determined via appropriate tests, with significance markers annotated (* *p* < 0.05, ** *p* < 0.01, *** *p* < 0.001).

To further validate the anti-inflammatory effects of Niudali observed via lung proteomics and functional network analyses, we assessed key inflammatory mediators in bronchoalveolar lavage fluid (BALF) via ELISA. Specifically, we quantified the levels of IgE and the type 2 cytokines IL-4, IL-5, and IL-6, which are central to driving allergic airway inflammation and eosinophilic responses in asthma patients. Consistent with the proteomic findings indicating immune modulation, both low- and high-dose nutritional treatments resulted in marked reductions in the levels of all four inflammatory markers compared with those in the asthma group (model group). Notably, high-dose Niudali led to a significant decrease in IL-5, IL-6, and IgE, with all comparisons showing strong statistical significance (*p* < 0.001). IL-4 levels were also significantly reduced (*p* = 0.019), indicating attenuation of Th2-skewed inflammation. Similarly, low-dose Niudali produced consistent and significant reductions in IL-5, IL-6, and IgE (all *p* < 0.001), along with a meaningful decrease in IL-4 (*p* < 0.01), albeit slightly less pronounced than that in the high-dose group. These results, illustrated in Figure 9, clearly demonstrate that Niudali not only reshaped the lung tissue proteome toward an anti-inflammatory state but also directly suppressed key soluble inflammatory mediators associated with asthma pathophysiology. The reduction in IgE, a hallmark of allergic sensitization, and the concurrent suppression of IL-4, IL-5, and IL-6 underscore Niudali's capacity to mitigate eosinophilic inflammation, dampen allergic immune responses, and promote the restoration of airway immune homeostasis. This integrated evidence from both tissue proteomics and BALF cytokine analysis strongly supports the conclusion that Niudali exerts its therapeutic effects through multilevel immunomodulation, targeting both cellular protein networks and soluble inflammatory drivers in allergic airway disease.

## Discussion

This study provides comprehensive proteomic evidence that Niudali (Millettia speciosa) exerts potent immunomodulatory effects on allergic asthma, primarily by suppressing eosinophilic inflammation and rewiring immunometabolic pathways. A key mechanistic insight is the profound downregulation of CCL24, a pivotal eosinophil chemoattractant. CCL24 synergizes with IL-5 to drive eosinophilic infiltration, IL-13 production, and airway hyperresponsiveness (AHR) (5, 9). The role of the CCL24‒CCR3 axis extends beyond chemotaxis, contributing to airway epithelial dysfunction, fibrosis, and glucocorticoid resistance (7, 8, 10). Notably, therapeutic inhibition of this axis has been shown to be effective in models of lung and skin fibrosis (7), and aberrant CCL24 production, such as through dendritic cell TPL2 deficiency, exacerbates Th2-driven airway inflammation (16). Our data demonstrate that Niudali effectively abolishes CCL24 expression, likely disrupting this inflammatory loop and reducing eosinophil-driven tissue remodeling.However, we acknowledge that proteomic analysis was conducted only on the high-dose Niudali group, and direct dose-dependent proteomic changes were not assessed. Nonetheless, both low- and high-dose treatments significantly reduced BALF levels of IgE, IL-4, IL-5, and IL-6, suggesting a dose-dependent effect at the level of soluble inflammatory mediators. Our subsequent future studies will include multi-dose proteomic analyses to explore dose-dependent molecular mechanisms.

Furthermore, recent findings implicate eosinophil extracellular traps (EETs) in perpetuating airway inflammation via epithelial CCL24 induction and ILC2 activation, forming a self-reinforcing inflammatory circuit (1, 8). Niudali's ability to suppress CCL24 may therefore not only reduce eosinophil recruitment but also halt this pathogenic feedback loop. The observed proteomic changes extend beyond classical inflammatory mediators. Notably, Niudali modulates key lipid transport and antioxidant proteins, particularly ApoA1, ApoE, and Alb, which play emerging roles in regulating immune responses, reducing oxidative stress, and maintaining epithelial integrity (17, 18). These shifts suggest that Niudali enhances lipid-based immune regulation, an increasingly recognized mechanism in airway homeostasis. Prior lipidomic studies in asthma models have revealed dysregulation of glycerophospholipid and sphingolipid metabolism (19), mirroring the lipid-related pathways enriched in our dataset, including cholesterol metabolism, phospholipid efflux, and PPAR signaling. This immunometabolic reprogramming is further supported by enriched antioxidant pathways and complement regulation. PPI network analysis revealed central hubs, such as Alb, Apoe, Proc, Serpina7, and Serpinf2, which coordinate lipid transport, coagulation balance, and immune resolution. These interconnected nodes indicate that Niudali acts not by modulating isolated proteins but by reshaping fundamental biological networks that govern inflammation and repair.

Interestingly, these molecular signatures are consistent with the emerging concept of the gut–lung axis, wherein gut-derived metabolites such as short-chain fatty acids (SCFAs) influence lung immunity. SCFAs have been reported to suppress Th2 cytokine responses, promote regulatory T cell expansion, and enhance epithelial barrier integrity (11). Prior studies have shown that Millettia speciosa enhances gut barrier integrity and alters microbiota composition (20), suggesting that part of the effect of Niudali may be mediated via microbiota-driven SCFA production. In the present study, although SCFA levels and gut microbiota composition were not directly measured, the enrichment of pathways related to lipid metabolism, antioxidant defense, and immune regulation observed in our proteomic data shows partial overlap with pathways previously associated with SCFA-mediated immunomodulation. Therefore, the potential involvement of the gut-lung axis in the therapeutic effects of Niudali should be interpreted with caution and remains speculative. Future studies integrating microbiome profiling and metabolomic analyses will be required to validate this hypothesis. Additionally, the enrichment of tryptophan metabolism, lipid-based immune pathways, and PPAR signaling echoes transcriptomic findings that highlight the systemic immunomodulatory effects of Millettia speciosa constituents, particularly isoflavones (13). This finding supports a multilayered therapeutic mechanism that integrates immune suppression, redox balance, and metabolic regulation, a hallmark of both modern systems biology and traditional Chinese medicine (TCM) frameworks.

Taken together, our findings complement growing TCM-based proteomics studies that leverage high-throughput platforms such as iTRAQ and LC‒MS/MS to decipher complex herbal mechanisms in respiratory diseases (18, 21, 22). The successful application of DIA proteomics in this study offers a robust framework not only for characterizing the effects of Niudali but also for guiding the development of microbiota-targeted, metabolic, or botanical interventions for asthma.

Our study demonstrated that Niudali (Millettia speciosa) exerts robust therapeutic effects on allergic asthma through the coordinated suppression of CCL24-driven eosinophilic inflammation and the rewiring of immunometabolic networks. This dual action, which targets both proinflammatory chemokines and lipid-antioxidant pathways, highlights the capacity of Niudali to modulate both immune and epithelial compartments. Importantly, the use of DIA-based proteomics enabled high-depth and reproducible quantification of lung tissue proteins, providing a systems-level perspective on the therapeutic mechanisms of Niudali. The integration of proteomic signatures with soluble mediator reductions (IgE, IL-4, IL-5, and IL-6) further confirms its impact on airway immune homeostasis. These findings establish a strong proteomic foundation for Niudali as a promising gut‒lung axis-modulating therapy for asthma.

But, this study has several limitations that should be acknowledged. First, although our proteomic data suggest potential involvement of immunometabolic pathways related to the gut-lung axis, we did not directly assess gut microbiota composition or measure short-chain fatty acid (SCFA) levels. Therefore, the proposed link between Niudali treatment and microbiota-derived metabolites remains speculative. Second, the sample size in this study was relatively limited, which may affect the generalizability of the findings. Third, our analysis was primarily based on lung tissue proteomics, and multi-omics integration (e.g. microbiome, metabolomics, and transcriptomics) was not performed. Future studies incorporating larger cohorts and multi-omics approaches will be essential to validate and extend these findings, particularly to elucidate the role of the gut-lung axis in the therapeutic effects of Niudali.

## Data Availability

The proteomics data generated in this study have been deposited in the iProX at iProX - integrated Proteome resources under accession number IPX0017373002.
