# Supplementary figures and images for: Millettia speciosa reprograms the lung proteome and suppresses CCL24-driven eosinophilic inflammation in allergic asthma
Source: Front Allergy. 2026 Jun 3;7:1726706. doi: 10.3389/falgy.2026.1726706 (PMC13272939; doi:10.3389/falgy.2026.1726706)

# Functional annotation of Proteins

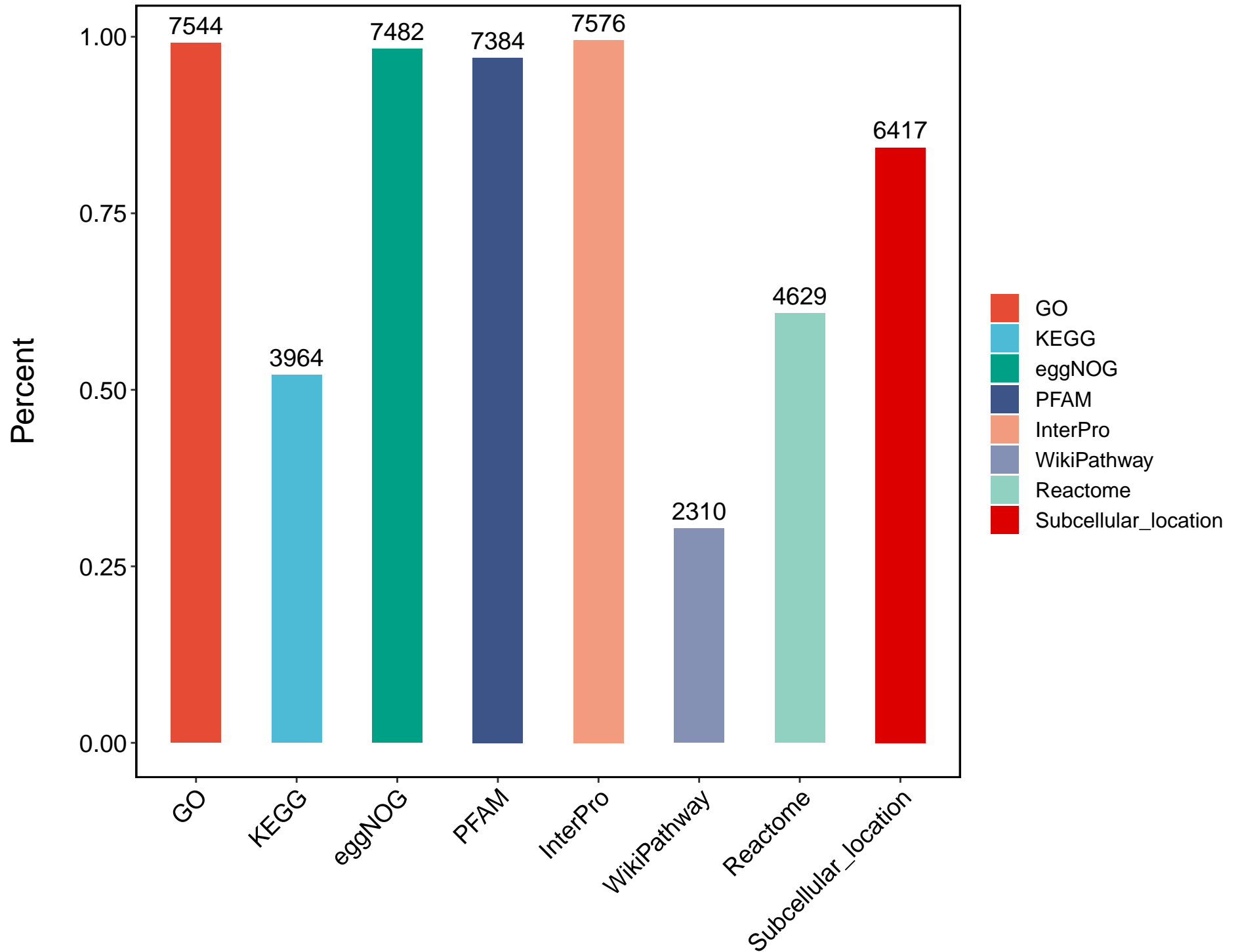

Supplement: Supplementary file 1 [file Datasheet1.pdf]

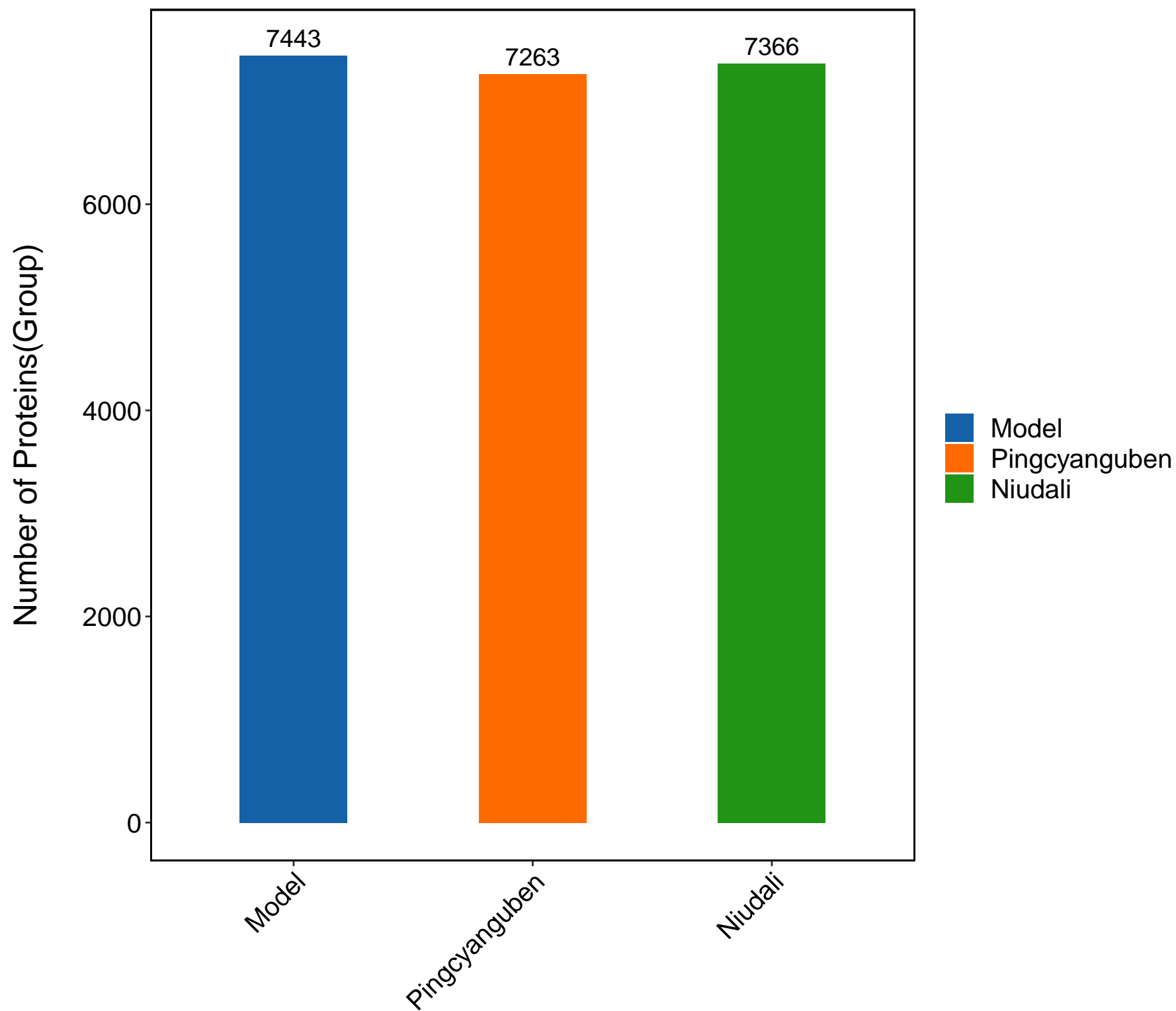

Supplement: Supplementary file 2 [file Datasheet2.pdf]

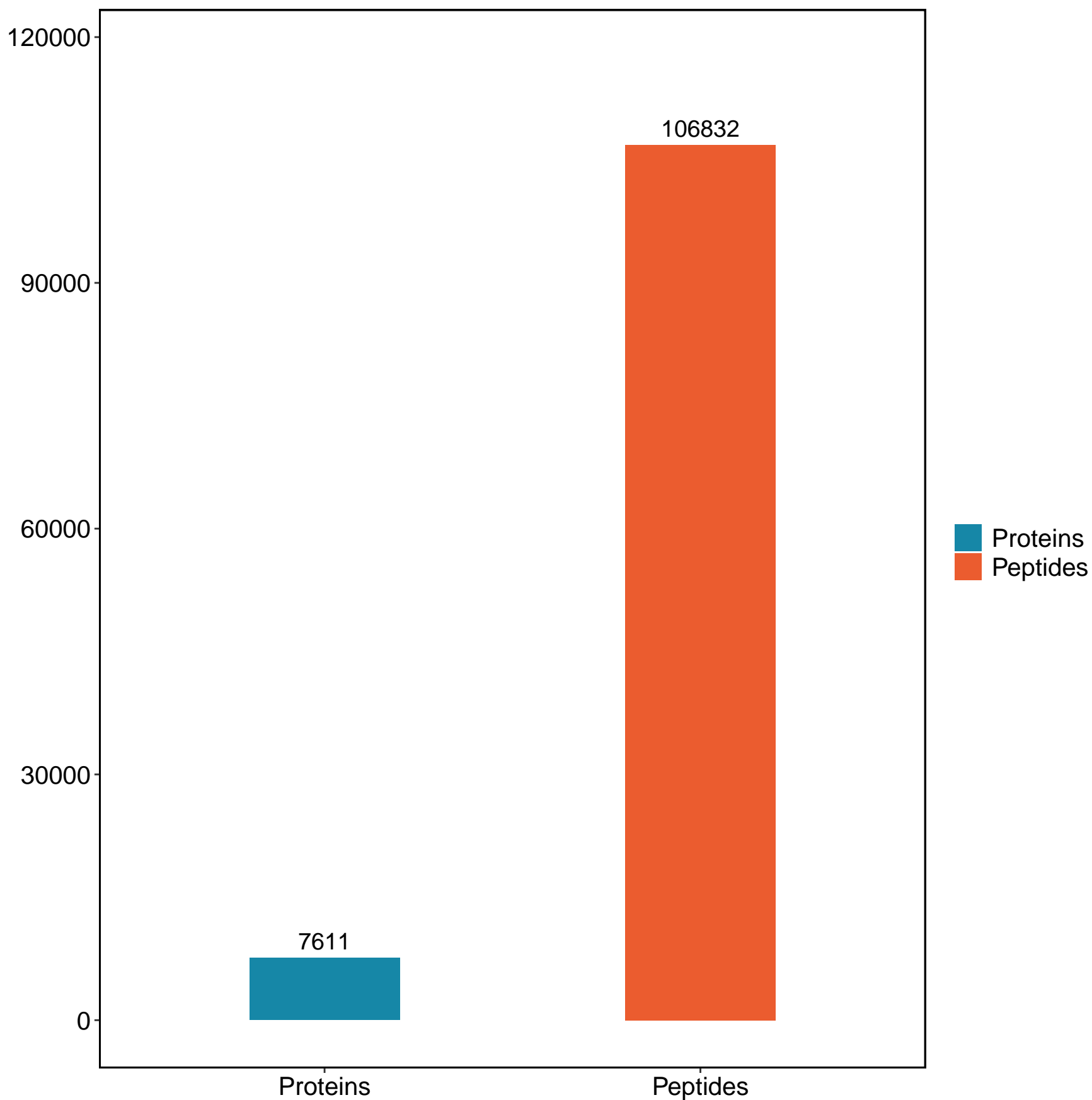

Supplement: Supplementary file 3 [file Datasheet3.pdf]

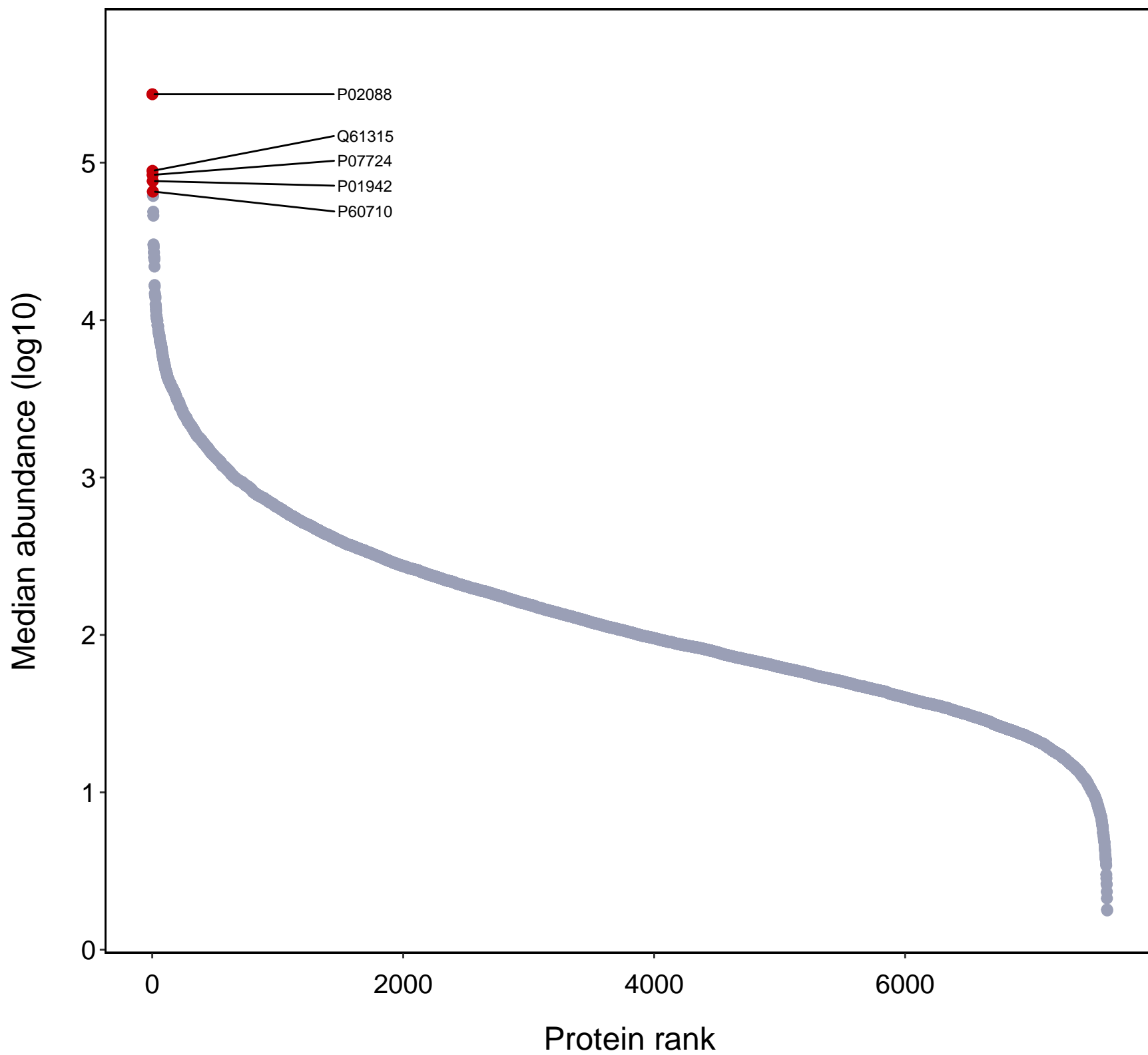

Supplement: Supplementary file 4 [file Datasheet4.pdf]

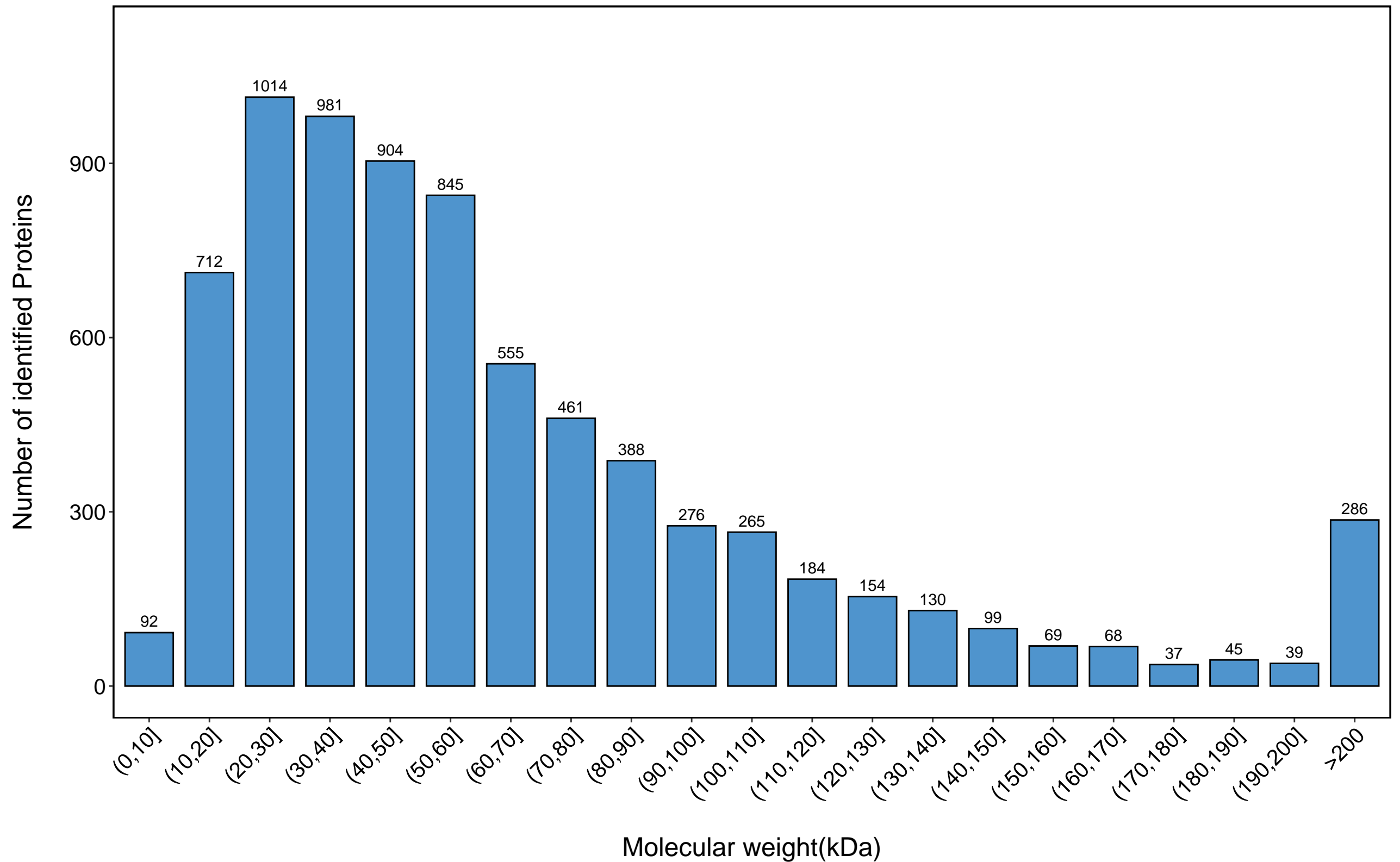

Supplement: Supplementary file 5 [file Datasheet5.pdf]

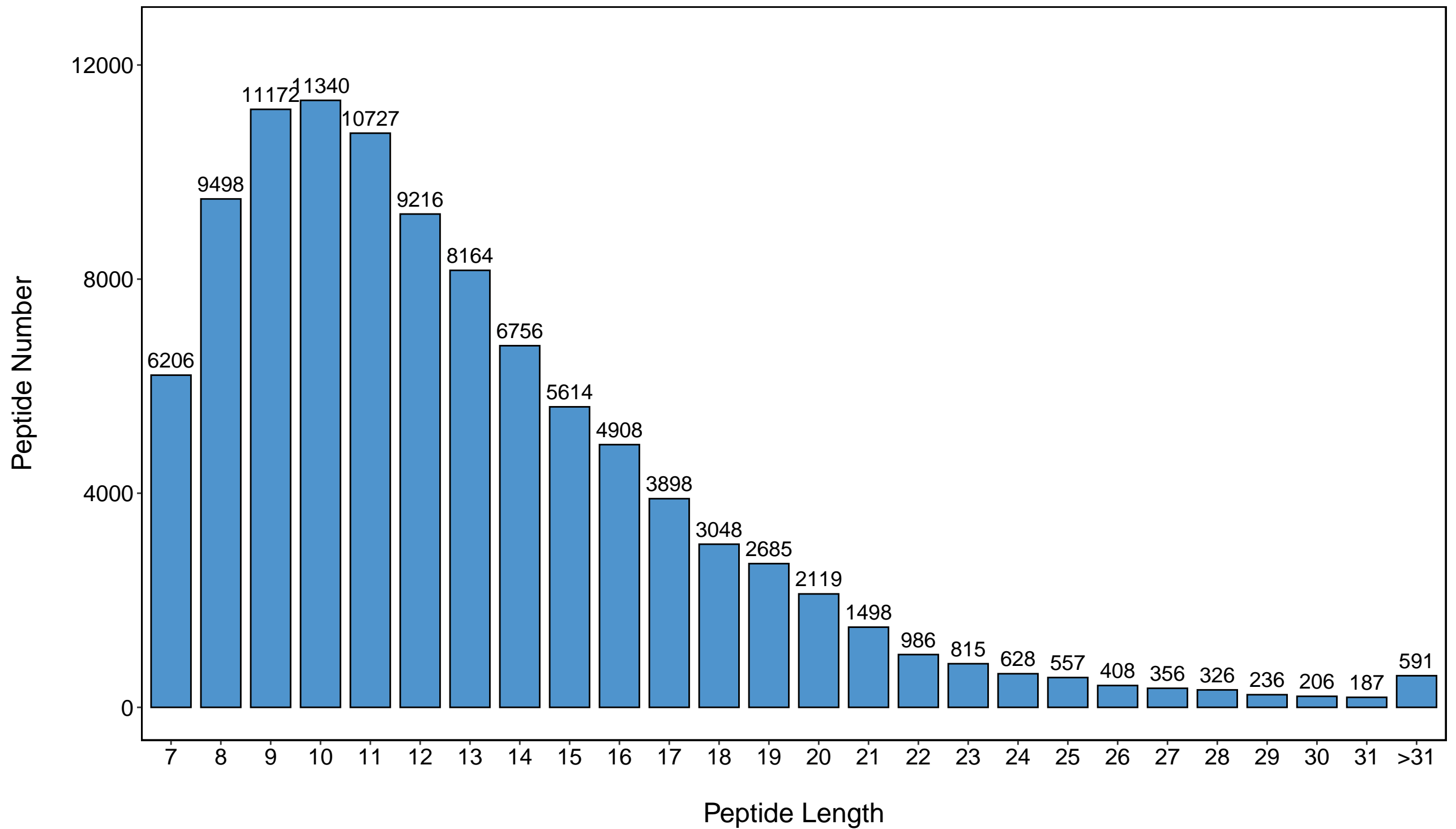

Supplement: Supplementary file 6 [file Datasheet6.pdf]

Number of identified proteins

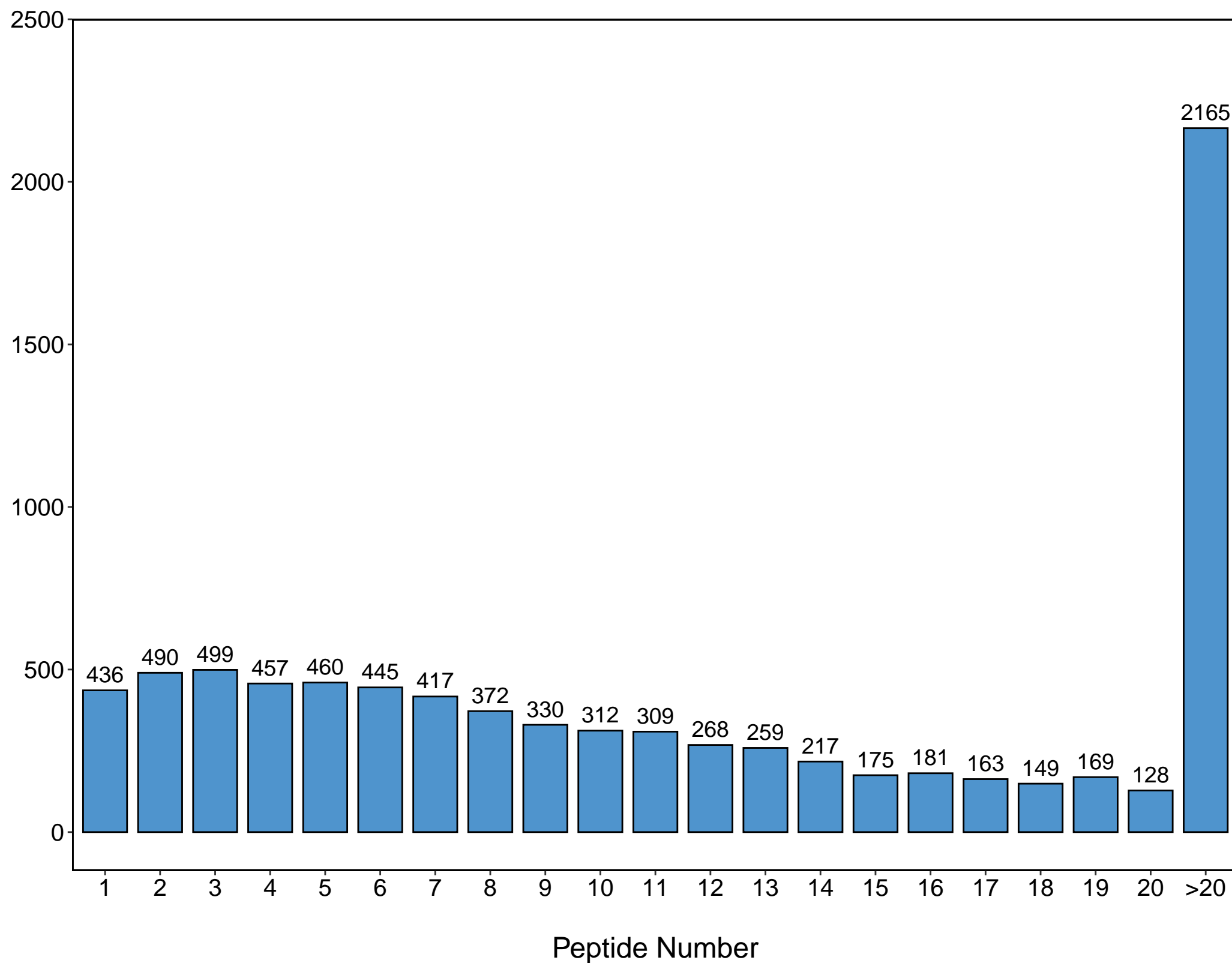

Supplement: Supplementary file 7 [file Datasheet7.pdf]

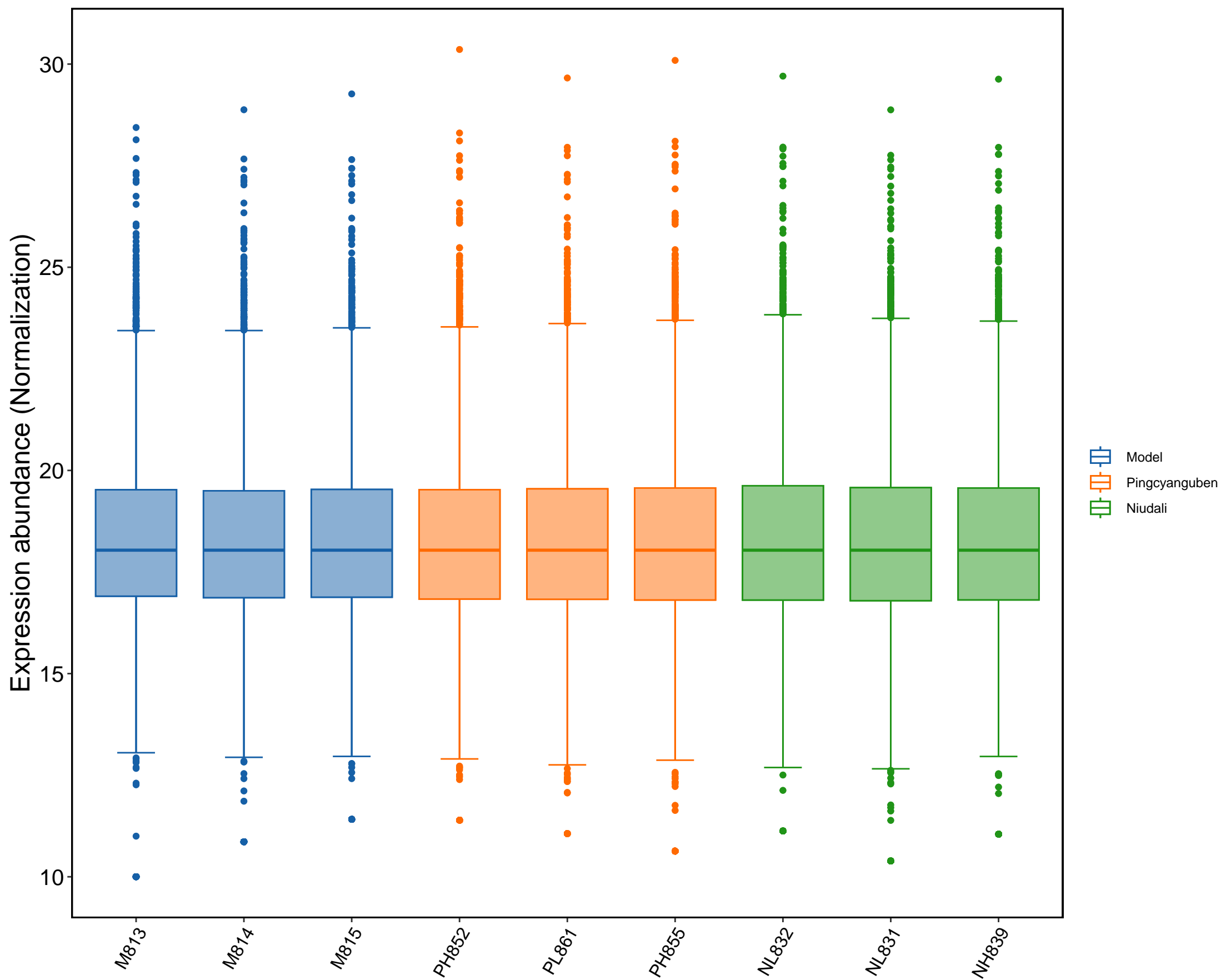

Supplement: Supplementary file 8 [file Datasheet8.pdf]

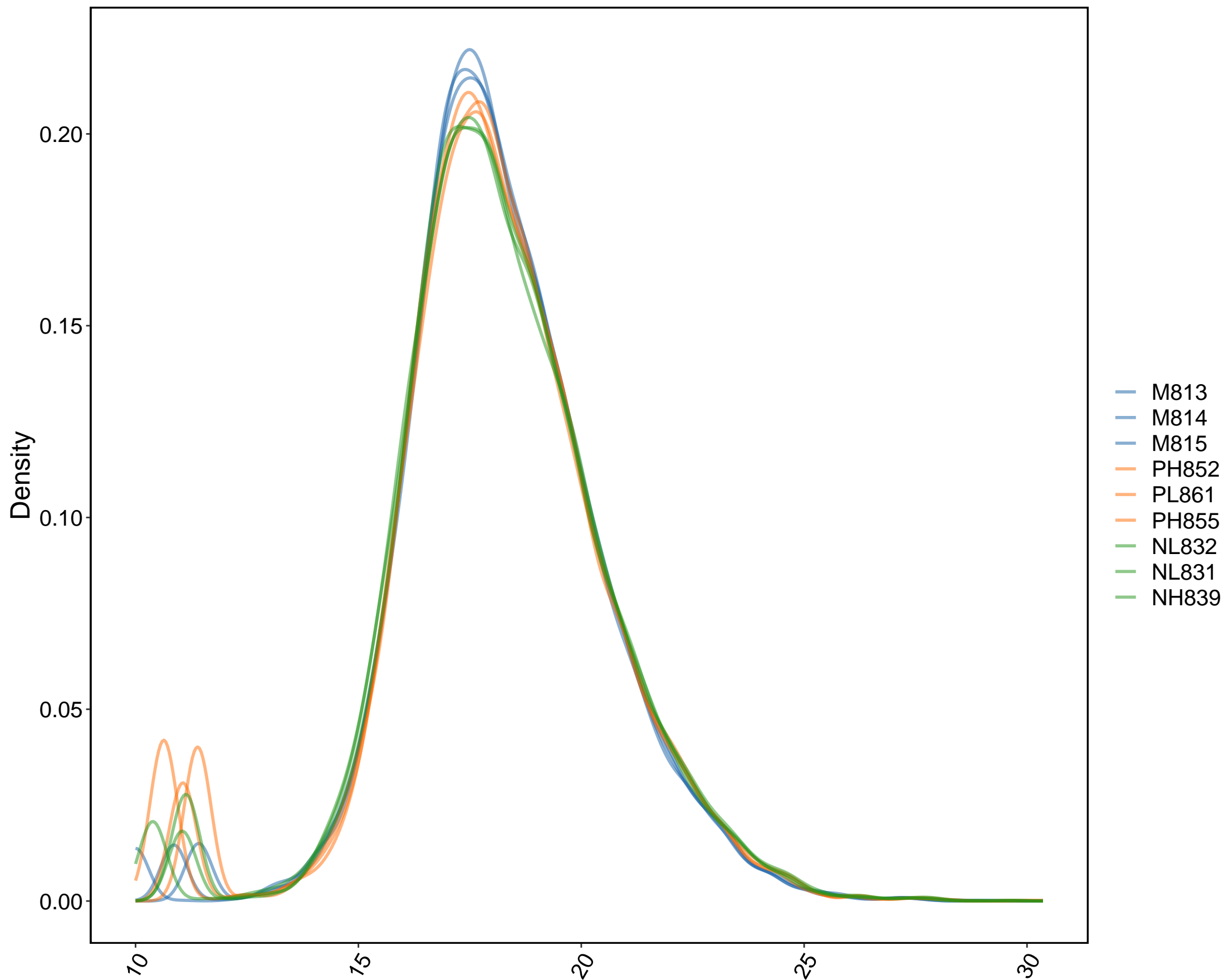

Supplement: Supplementary file 9 [file Datasheet9.pdf]

# PCA

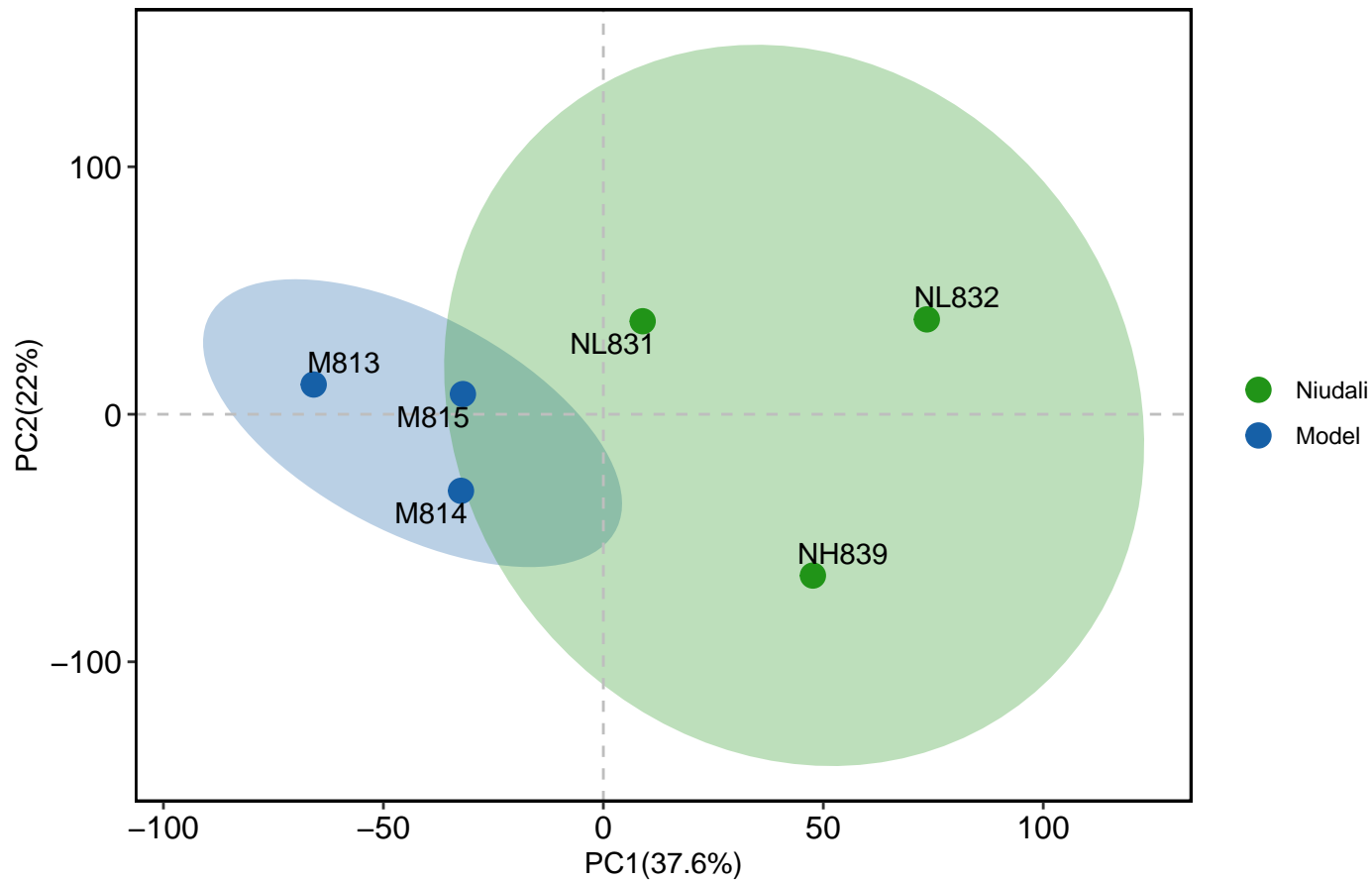

Supplement: Supplementary file 10 [file Datasheet10.pdf]

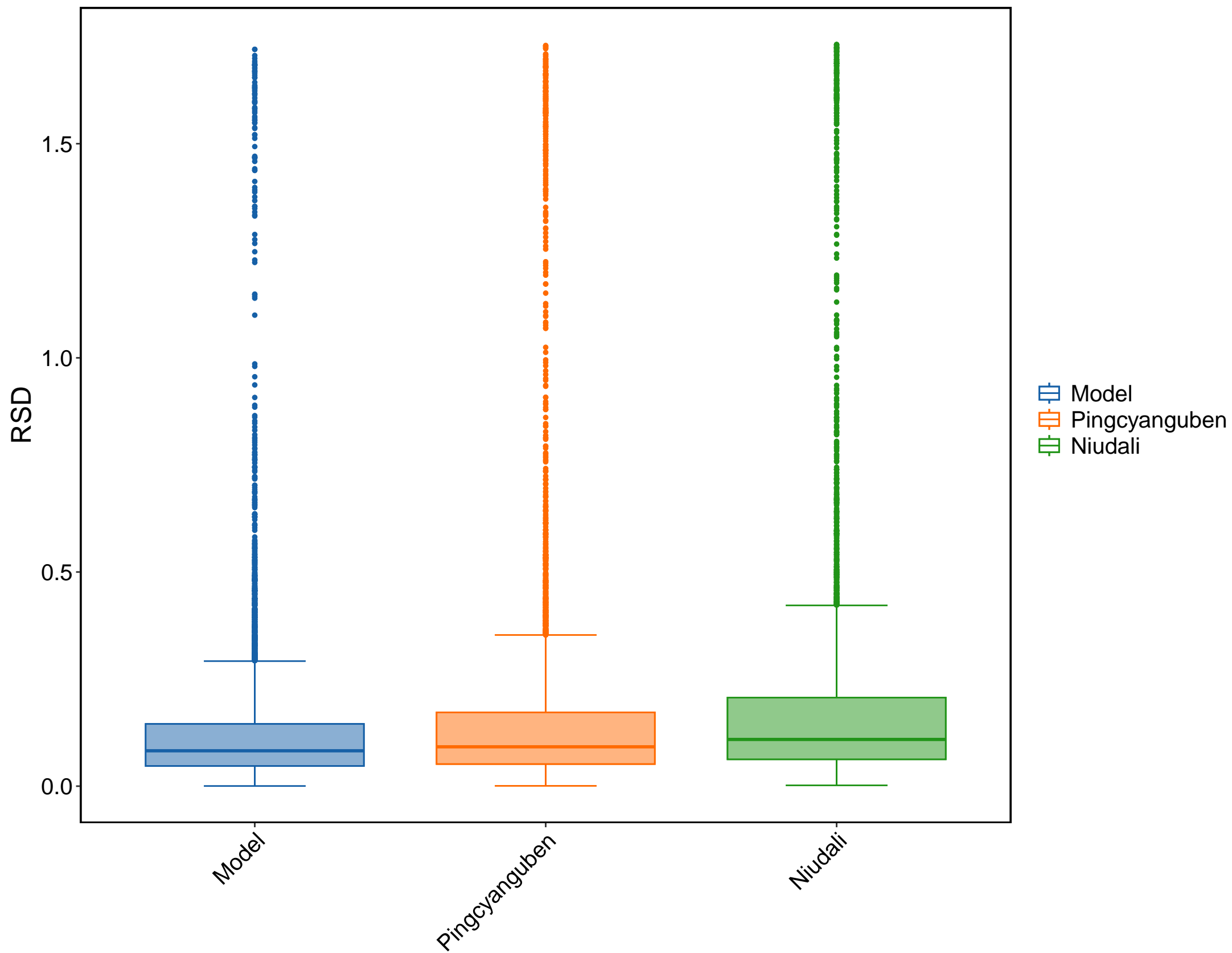

Supplement: Supplementary file 11 [file Datasheet11.pdf]
