# Supplementary material for: Millettia speciosa reprograms the lung proteome and suppresses CCL24-driven eosinophilic inflammation in allergic asthma
Source: Front Allergy. 2026 Jun 3;7:1726706. doi: 10.3389/falgy.2026.1726706 (PMC13272939; doi:10.3389/falgy.2026.1726706)
Supplement: Supplementary file 12 [file Table1.docx]

**eFigure
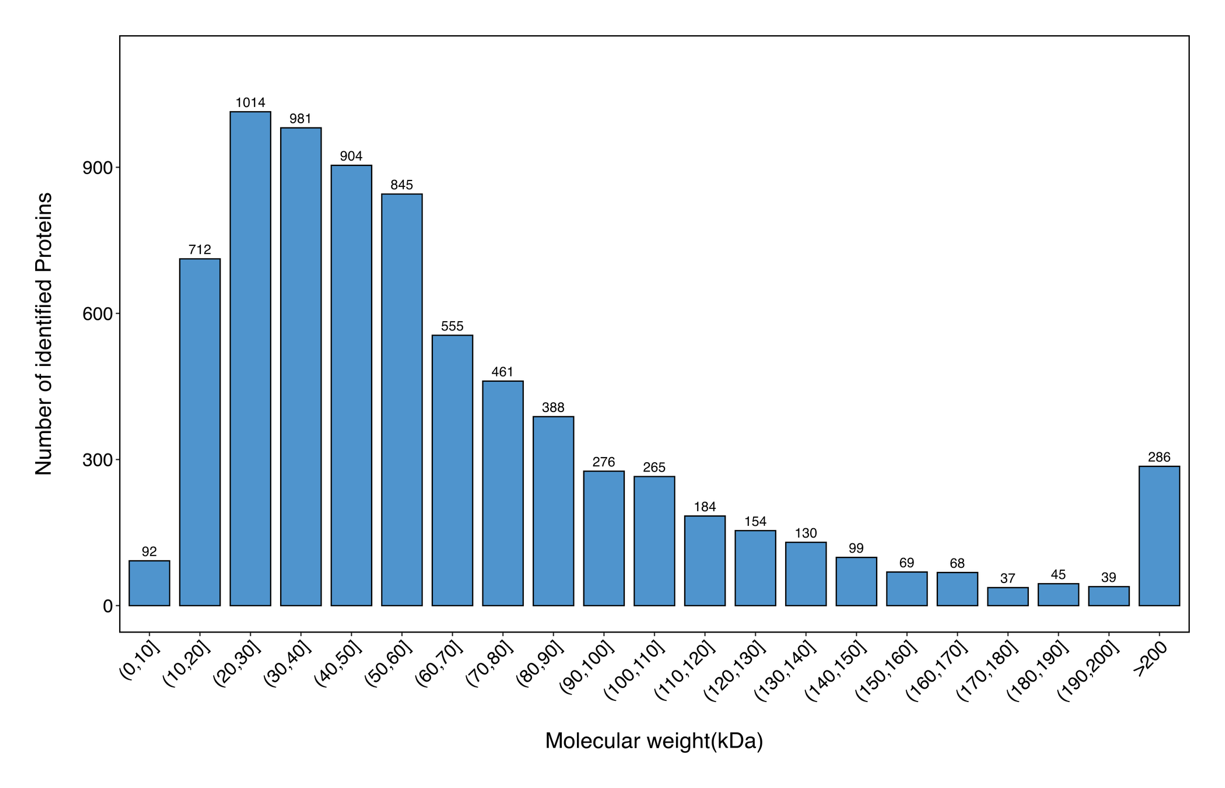
 1**

**eFigure.1 Distribution of Identified Proteins by Molecular Weight in Lung Proteome.** Bar plot representing the distribution of identified proteins across molecular weight bins in the lung proteome dataset. The x-axis indicates molecular weight ranges (kDa), while the y-axis shows the number of identified proteins within each range.

**
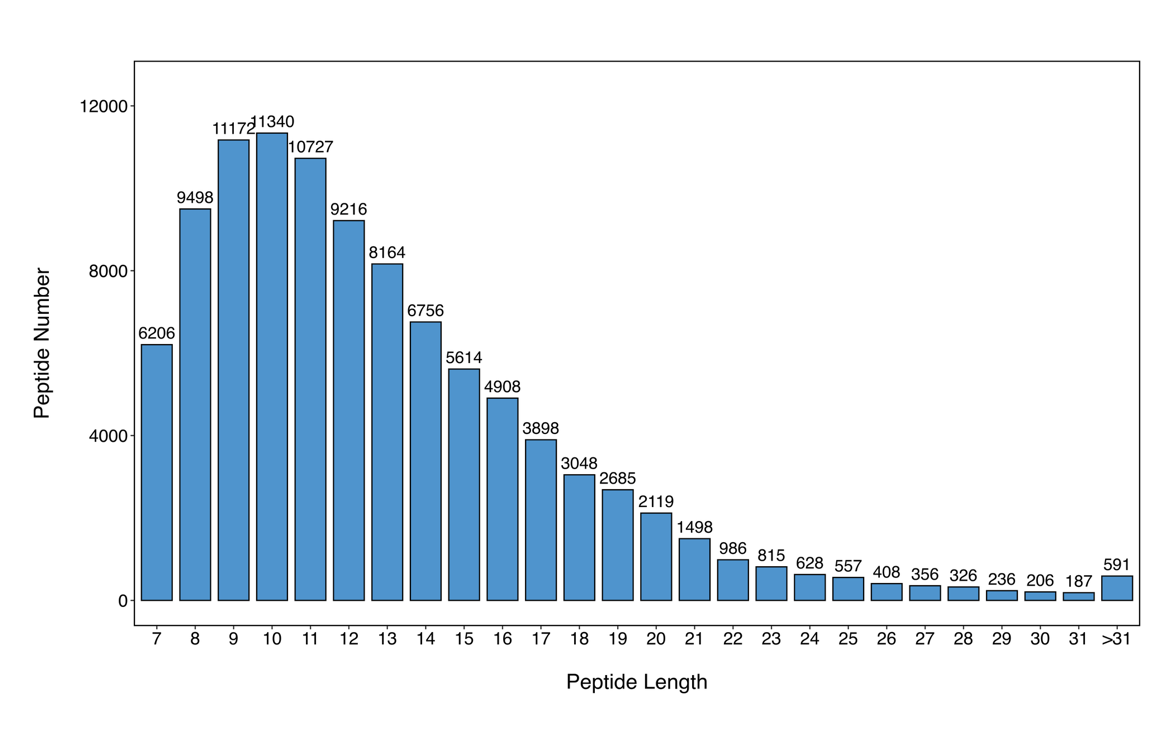
eFigure 2**

**eFigure.2 Bar plot showing the distribution of identified peptides based on peptide length.** The x-axis represents peptide length (number of amino acids), while the y-axis indicates the number of peptides. The majority of peptides are distributed between 9 to 11 amino acids, peaking at 10 amino acids (13,440 peptides), which is characteristic of efficient tryptic digestion.

**eFigure 3**


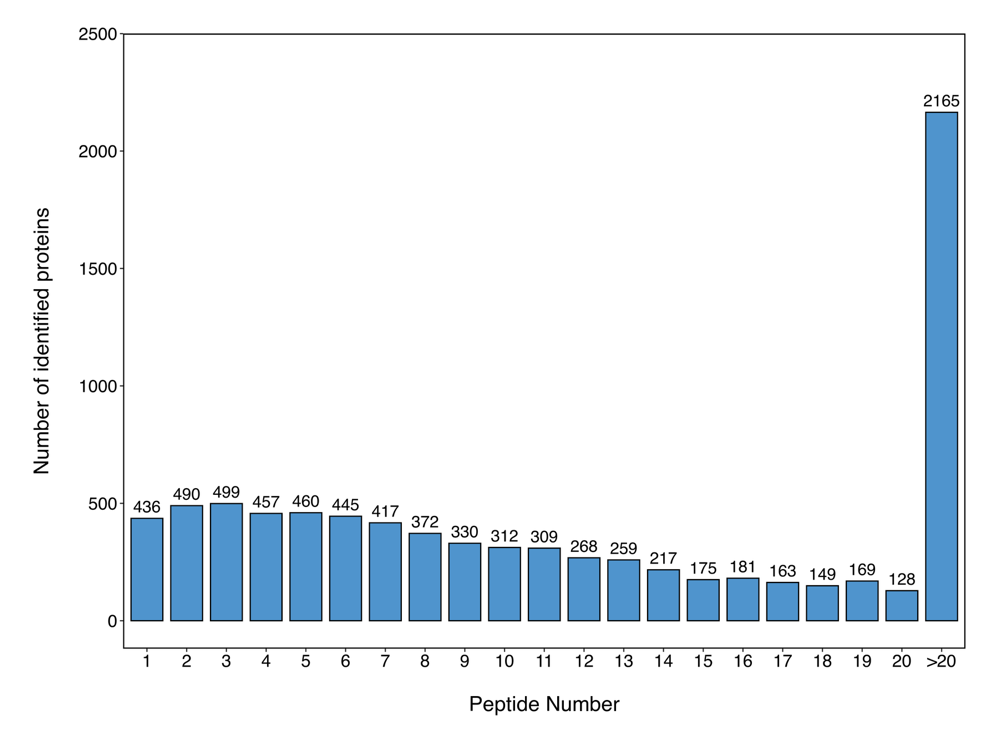


**eFigure.3 Peptide Coverage Distribution of Identified Proteins in Lung Proteome.** Bar plot representing the distribution of identified proteins based on the number of unique peptides mapped per protein. The x-axis indicates peptide count per protein, and the y-axis shows the number of proteins identified.

**eFigure 4**

**
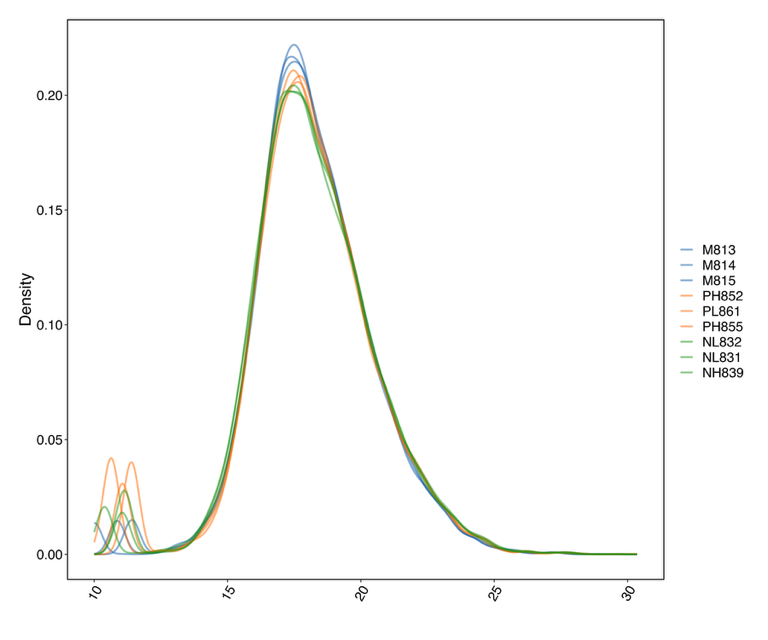
**

**eFigure.4 Density Distribution of Protein Intensities Across Samples.** Density plot showing the distribution of log-transformed protein intensities across all biological replicates and groups. The x-axis represents log-transformed protein intensity, while the y-axis represents density.

**eFigure 5
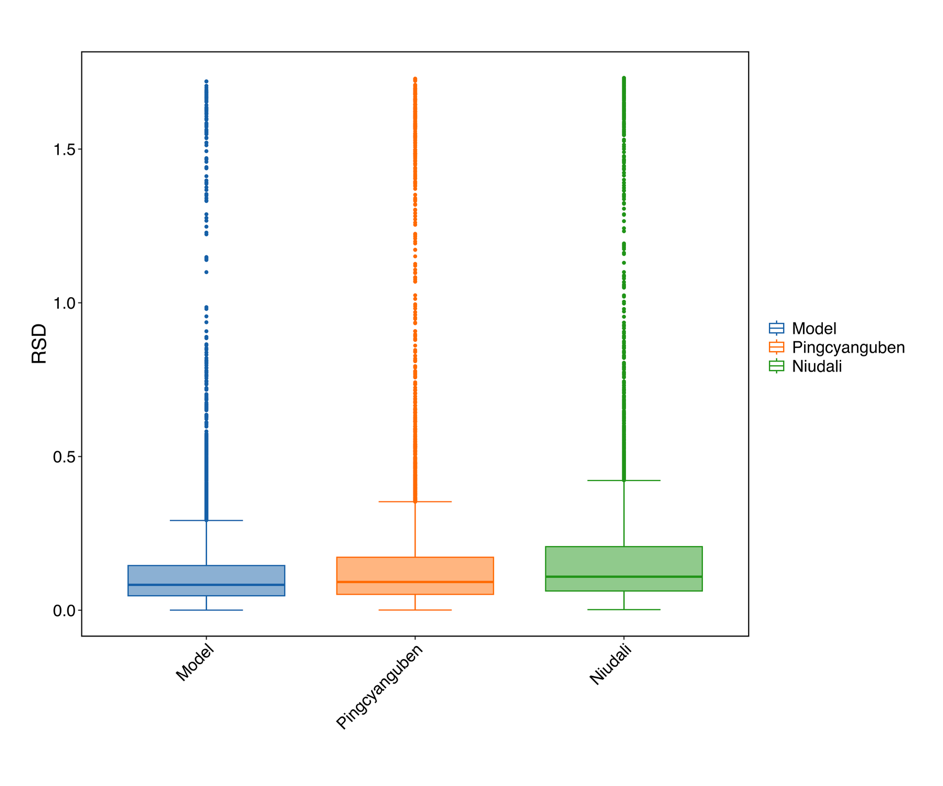
**

**eFigure.5 Boxplot of RSD Values Across Treatment Groups.** Boxplot displaying the distribution of relative standard deviation (RSD) values for protein intensities within each treatment group: Model (blue), Pingyangguben (orange), and Niudali (green). The x-axis indicates the treatment groups, while the y-axis represents RSD values, which quantify the variability among biological replicates within each group

**eFigure 6**

**
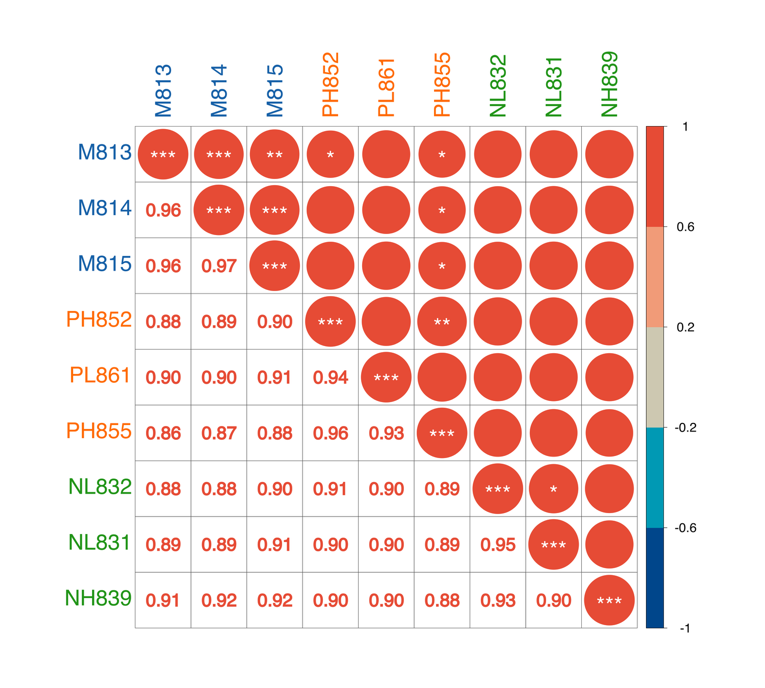
**

**eFigure.6 Correlation Matrix of Protein Intensities Across Samples.** Correlation matrix depicting Pearson correlation coefficients between biological replicates from the Model (blue), Pingyangguben (orange), and Niudali (green) groups. The color gradient and circle size represent the strength and direction of correlation, with values ranging from -1 (perfect negative) to +1 (perfect positive), as indicated by the color bar. Higher correlation values (closer to 1) are represented by larger, deeper red circles, indicating strong similarity in proteomic profiles within and across groups. Asterisks denote significance levels (* p < 0.05, ** p < 0.01, *** p < 0.001).
